# A *ptsH* mutation suppresses growth defects and antibiotic sensitivity in a *cpgA* mutant defective in metabolite proofreading

**DOI:** 10.1128/jb.00162-25

**Published:** 2025-08-14

**Authors:** Ankita J. Sachla, Ahmed Gaballa, Diana Herrera, John D. Helmann

**Affiliations:** 1 Department of Microbiology, Cornell University 5922, Ithaca, New York, USA; The Ohio State University, Columbus, Ohio, USA

**Keywords:** histidine protein (HPr), glyceraldehyde-3-phosphate dehydrogenase (GAPDH), allosteric regulation, promiscuous enzyme, ribosome assembly GTPase, cefuroxime, moenomycin, fosfomycin, CRISPR

## Abstract

*Bacillus subtilis* CpgA (circularly permuted GTPase) is a ribosome assembly GTPase that has a secondary function as a metabolite proofreading enzyme. CpgA hydrolyzes 4-phosphoerythronate, a toxic metabolite produced from erythrose-4-phosphate by glyceraldehyde-3-phosphate dehydrogenase (GAPDH). In a ∆*cpgA* strain, carbon sources that feed into the pentose phosphate pathway trigger metabolic intoxication. This results in poor growth and increased sensitivity to antibiotics that block peptidoglycan synthesis, a process reliant on sugars from central metabolism. Here, we describe a mutation in *ptsH* (*ptsH*-G54D) that improves growth of a ∆*cpgA* strain on media containing both glucose and gluconate. The *ptsH* gene encodes the histidine-containing phosphocarrier protein (HPr) that functions in phosphotransferase system sugar import and gene regulation. Prior studies of HPr suggested three possible mechanisms to account for the ability of HPr-G54D to increase fitness of the ∆*cpgA* strain: (i) restricting HPr-dependent uptake of glucose, (ii) reducing the GAPDH-dependent production of 4-phosphoerythronate, or (iii) decreasing expression of genes required for uptake and catabolism of gluconate. Here, we present evidence consistent with the third model: HPr-G54D improves fitness of a ∆*cpgA* strain by increasing catabolite repression of the gluconate operon. Consistently, genetic suppression by HPr-G54D requires Ser46, a site of regulatory phosphorylation important for carbon catabolite repression. In addition, we demonstrate that the metabolic proofreading function of CpgA is conserved among related gram-positive bacteria.

**IMPORTANCE:**

Metabolism relies on the concerted action of hundreds of enzymes, many of which have some activity with non-canonical substrates. The resulting reactions constitute an often-ignored underground metabolism. Glyceraldehyde-3-phosphate dehydrogenase catalyzes a secondary reaction that produces 4-phosphoerythronate, a toxic dead-end metabolite. *Bacillus subtilis* CpgA is a widely conserved metabolite proofreading enzyme that protects cells against metabolic intoxication, which can increase antibiotic sensitivity. Loss of CpgA can be suppressed by an altered function mutation affecting the histidine-containing phosphocarrier protein (HPr). This mutant HPr protein increases carbon catabolite repression to restrict import of intoxicating gluconate. These studies highlight the ability of mutations in HPr to rewire carbon catabolism to help avoid the toxic effects of metabolic dysregulation.

## INTRODUCTION

*Bacillus subtilis* CpgA (circularly permuted GTPase) is a small, ribosome-associated GTPase that functions during assembly of the small (30S) ribosomal subunit (1, 2). A *cpgA* null mutant (∆*cpgA*) has diverse phenotypes, including some consistent with a defect in ribosome assembly (3). The *Escherichia coli* CpgA ortholog RsgA also functions in late stages of 30S assembly. RsgA displaces a folding chaperone (RbfA) to allow entry of newly synthesized 30S subunits into the pool of translating ribosomes (4).

**Fig 1:**
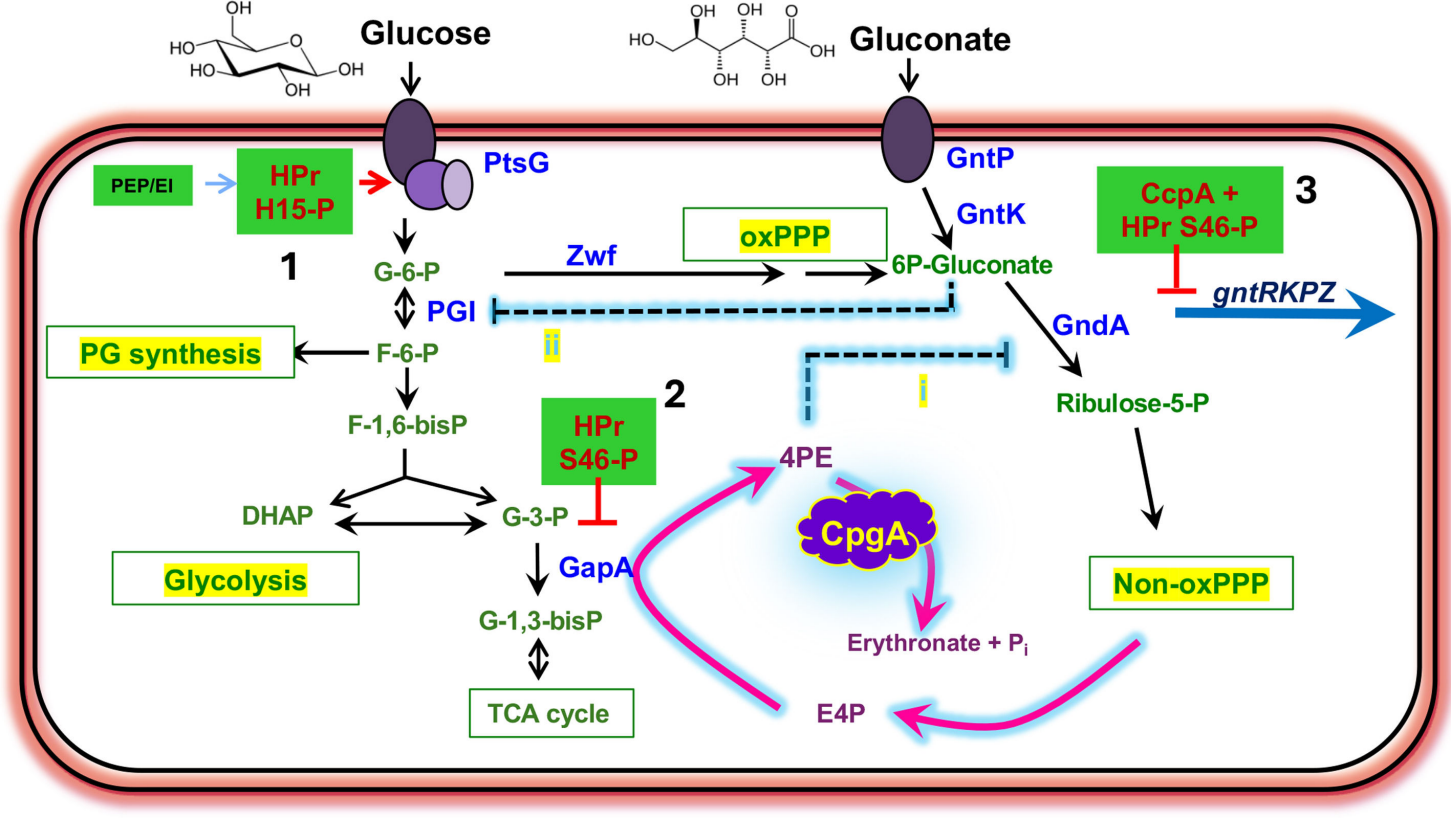
Possible roles of histidine-containing phosphocarrier protein (HPr)-G54D in mitigating metabolite intoxication in the ∆*cpgA* mutant. The ∆*cpgA* mutant strain is sensitive to growth inhibition by glucose and gluconate. Both glucose and gluconate are catabolized, at least in part, through the PPP. Glucose imported by the phosphotransferase system protein PtsG enters as glucose-6-phosphate (G-6-P), which is partitioned into glycolysis by Pgi or into the oxidative phase of the PPP (oxPPP) by glucose-6-phosphate dehydrogenase (Zwf). The toxic metabolite 4PE is produced by a promiscuous reaction of GapA with E4P, an intermediate in the non-oxidative phase of the pentose phosphate pathway (non-oxPPP). Accumulation of 4PE is normally prevented by the action of CpgA. In a ∆*cpgA* mutant, 4PE accumulates and inhibits 6-phosphogluconate dehydrogenase (GndA) (step i). Accumulation of 6-PG competitively inhibits Pgi, leading to metabolic gridlock (step ii). The *ptsH*-G54D allele suppresses metabolic intoxication in media containing glucose and gluconate. We here test three possible mechanisms for this effect (green boxes): (1) reduction of glucose import, (2) inhibition of GapA-dependent production of 4PE (possibly by increasing enzyme fidelity), or (3) increased carbon catabolite repression of gluconate import and catabolism.

Apart from its role in ribosome assembly, we previously demonstrated that CpgA has an additional moonlighting function. CpgA dephosphorylates 4-phosphoerythronate (4PE), a toxic metabolite that inhibits central carbon metabolism (5). In mammals, 4PE is generated by a promiscuous reaction of glyceraldehyde-3-phosphate dehydrogenase (GAPDH) with erythrose-4-phosphate (E4P), a pentose phosphate pathway (PPP) intermediate (6). In *Bacillus subtilis*, elevated levels of 4PE inhibit 6-phosphogluconate dehydrogenase (GndA) and the resulting accumulation of 6-phosphogluconate inhibits phosphoglucoisomerase (Pgi) (5) (Fig. 1). In ∆*cpgA* mutants, this cascade of enzyme inhibition results in numerous media-dependent phenotypes: small colony size on lysogeny broth (LB) agar, growth inhibition by glucose and gluconate, altered cell wall synthesis, aberrant morphology, and sensitivity to peptidoglycan synthesis inhibitors (2, 5, 7, 8). All these phenotypes result largely from defects in carbon metabolism. In addition, Δ*cpgA* mutants are more sensitive to antibiotics that target the ribosome and are cold-sensitive for growth. These phenotypes result from defects in ribosome assembly (3, 5).

The strong growth inhibition on glucose and gluconate and the antibiotic sensitivity of ∆*cpgA* strains provide a powerful selection for suppressors. Previously, we found that selection for cefuroxime resistance (CEF^R^) on LB medium led to a null mutation in *ptsG* that prevents glucose import. We next selected for strains resistant to glucose intoxication and recovered hypomorphic alleles of glucose-6-phosphate dehydrogenase (*zwf*) that restrict the flux of glucose-6-phosphate into the PPP (Fig. 1). Similarly, *gntP* mutations that block gluconate import arise as suppressors of gluconate intoxication (5). We then selected for growth of the ∆*cpgA* strain in the presence of both glucose and gluconate, reasoning that the presence of two intoxicating carbon compounds would reduce the prevalence of mutations that restrict import. With this selection, one suppressor (*cpgA*.11) had mutations in both *zwf* and *ptsH*. The *ptsH* gene encodes the multifunctional histidine-containing phosphocarrier protein (HPr) (5).

HPr serves as the cytosolic, energy-coupling component of sugar import phosphotransferase systems (PTS). In this role, HPr is transiently phosphorylated on His15 (by enzyme I; PtsI). In response to elevated levels of glycolytic intermediates, HPr is phosphorylated on Ser46 by the bifunctional HPrK (HPr kinase/phosphatase) (9, 10), and this modified form is a poor substrate for phosphorylation by enzyme I (11). HPr-S46-P functions as a co-repressor with CcpA to mediate carbon catabolite repression (CCR) (9, 12). In addition, HPr-S46-P has been previously shown to bind to GapA and inhibit its activity *in vitro* (13).

We here characterize a *ptsH*-G54D mutation that encodes an altered function HPr (HPr-G54D) that partially rescues the poor growth and antibiotic sensitivity of ∆*cpgA* in the presence of glucose and gluconate. We provide evidence that HPr-G54D functions by increasing the CCR of the gluconate operon. We additionally show that CpgA orthologs from other Firmicutes (but not the *E. coli* ortholog RsgA) complement the ∆*cpgA* mutant and therefore likely retain this secondary, metabolite proofreading role.

## RESULTS AND DISCUSSION

### Possible roles of HPr-G54D in mitigating metabolite intoxication in the ∆*cpgA* mutant

**Fig 2:**
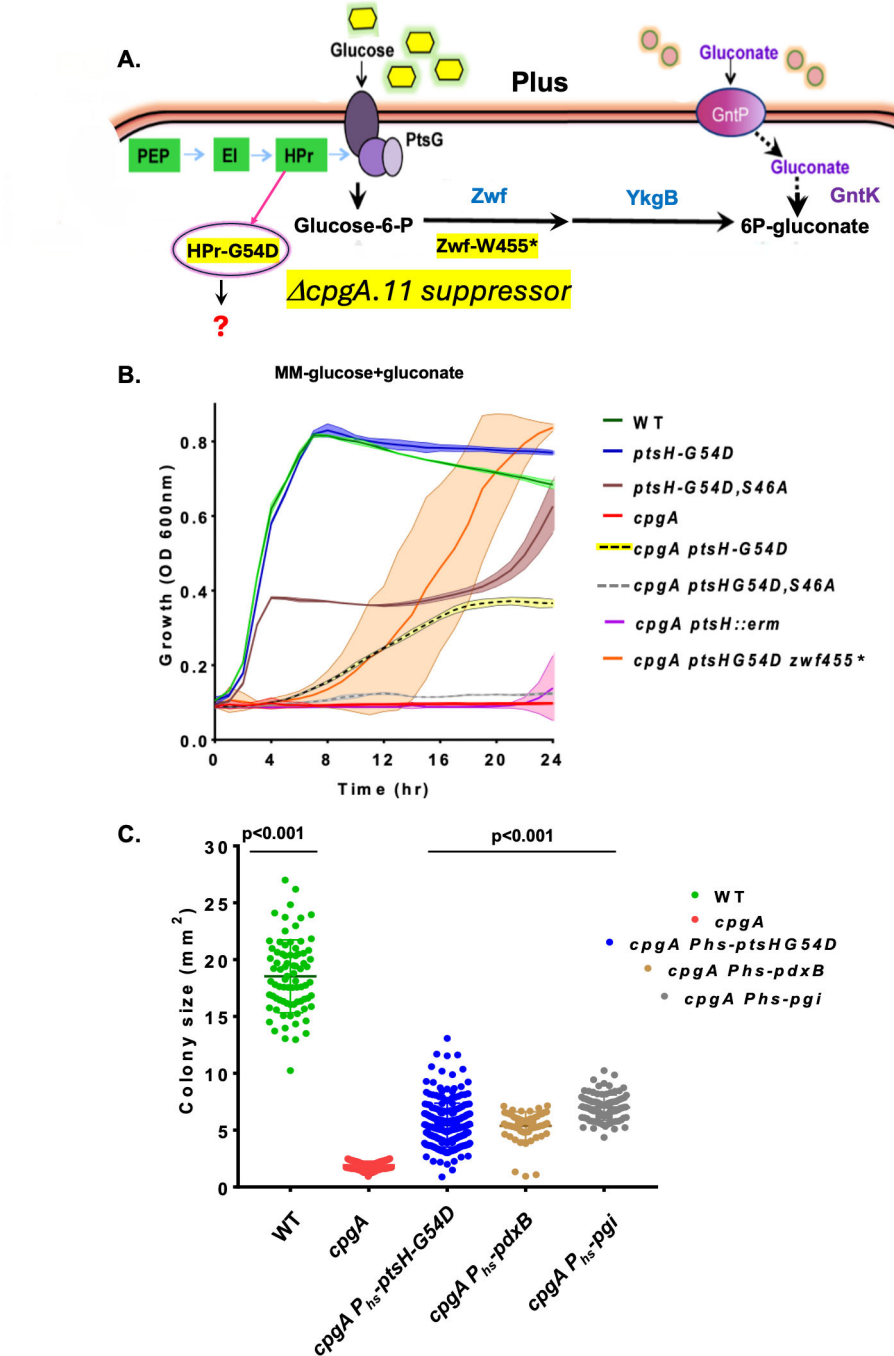
*ptsH*-G54D improves growth of ∆*cpgA***.** (**A**) Growth of a ∆*cpgA* strain with both glucose and gluconate led to a suppressor strain (*cpgA*.11) that carries mutations that reduce Zwf activity (W455*) and alter HPr (*ptsH*-G54D). (**B**) Aerobic growth of the indicated strains at 37°C in minimal medium (MM) supplemented with 0.5% glucose plus 0.5% gluconate as monitored by measuring optical density (OD_600_) over 24 h in a plate reader. The originally recovered ∆*cpgA.11* suppressor (orange) and the ∆*cpgA* strain carrying only the *ptsH*-G54D change (HBAS1855; yellow highlight) both grow in this medium, whereas the parent *cpgA::erm* strain (HB20401) does not (red). Growth is not restored by either *ptsH::erm* (HB20471; purple) or *ptsH*-S46A, G54D (HBAS1838; gray dashed line) mutations. (**C**) Colony size on LB plates as measured using ImageJ software (*n* > 68). The P_hyperspank_ (P_hs_)-constructs were tested in the presence of 0.5 mM of isopropyl β-D-1-thiogalactopyranoside (IPTG) and allowed expression of *ptsH*-G54D (HB24765), *pdxB* (HB24167), or *pgi* (HB1549). Two-way analysis of variance for multiple comparison was performed with Tukey’s post-correction. We show all comparisons with ∆*cpgA* to those of the other strains with *P* < 0.001.

The growth defect of Δ*cpgA* in the presence of glucose, gluconate, or the combination of the two carbon sources is due to the accumulation of the toxic metabolite 4PE (5) (Fig. 1). The original *cpgA*.11 suppressor strain was selected for improved growth in Mueller-Hinton (MH) medium containing both glucose and gluconate and carries two mutations: *zwf*-W455stop (affecting glucose-6-phosphate dehydrogenase) and *ptsH*-G54D (affecting HPr) (Fig. 2A). The *zwf*-W455stop mutation also arose in Δ*cpgA* strains selected only on glucose and was previously shown to reduce the activity of glucose-6-phosphate dehydrogenase by ~85% (5). Previous studies reveal that in M9 minimal medium, 43 ± 10% of imported glucose is partitioned into the PPP (14). Mutations in *zwf* likely function by restricting the flux of glucose-6-phosphate into the PPP and thereby reducing the adventitious synthesis of 4PE (5). The role of *ptsH*-G54D in preventing metabolite intoxication is less clear.

We identified three possible mechanisms that might explain how the *ptsH*-G54D mutation reduces intoxication by glucose plus gluconate (Fig. 1). We hypothesized that the *ptsH*-G54D mutation might (i) reduce PTS-dependent glucose import, (ii) decrease production of 4PE from erythrose-4-phosphate, or (iii) increase CCR to restrict import and catabolism of gluconate.

### The *ptsH*-G54D mutation improves growth of ∆*cpgA*

In minimal medium with both glucose and gluconate as carbon sources, the *cpgA::erm* strain (hereafter, Δ*cpgA*) was unable to grow, but the *cpgA*.11 suppressor grew to levels comparable to wild-type (WT), albeit after an extended lag phase of ~8 h (Fig. 2B). To test the effect of the *ptsH*-G54D in a strain lacking the *zwf*-W455stop mutation, we recreated this mutation using Clustered Regularly Interspaced Short Palindromic Repeats (CRISPR)-based mutagenesis in both the WT and ∆*cpgA* backgrounds. Like the original *cpgA*.11 suppressor strain, the ∆*cpgA* strain carrying only *ptsH*-G54D grew after a lengthy lag phase but with a reduction in overall cell yield (Fig. 2B). Thus, the *ptsH*-G54D mutation is beneficial to ∆*cpgA* cells, and the additional *zwf*-W455stop mutation further improves overall fitness. In contrast, a ∆*cpgA ptsH::erm* strain was unable to grow in this medium, suggesting that *ptsH*-G54D is an altered function mutation.

Consistent with the hypothesis that *ptsH*-G54D encodes an altered function protein, expression of HPr-G54D from an isopropyl β-D-1-thiogalactopyranoside (IPTG)-inducible promoter significantly increased the colony size of a ∆*cpgA* strain on LB medium (Fig. 2C). In contrast, induction of WT HPr from the same IPTG-inducible promoter was deleterious (Fig. S1). The observed increase in colony size with induction of HPr-G54D is comparable to that reported previously for induction of Pgi (Fig. 2C), the ultimate target of metabolic intoxication in ∆*cpgA* cells (5). Growth was also similarly increased by expression of *E. coli* PdxB, a paralog of GAPDH that functions as an E4P dehydrogenase (Fig. 2C). Thus, removing the metabolic block caused by 4PE (Pgi induction) or reducing the level of 4PE (PdxB induction) both benefit *cpgA* mutant cells. Although the *ptsH*-G54D mutation increases growth of ∆*cpgA* on LB medium, it does not suppress the cold sensitivity of the ∆*cpgA* strain (Fig. S2). This is expected since the cold-sensitive phenotype results from the separate role of CpgA in ribosome assembly rather than in countering metabolic intoxication.

### HPr-G54D still supports PTS-dependent sugar import

PTS-dependent sugar import relies on enzyme I (PtsI), which uses phosphoenolpyruvate to phosphorylate HPr on His15. HPr-H15-P then transfers this phosphate to the multidomain, sugar-specific enzyme II. When growing with glucose, the phosphorylated enzyme II (PtsG or EII^Glc^) transfers phosphate to the incoming sugar to generate glucose-6-phosphate. Since glucose is imported by both PTS-dependent and PTS-independent pathways (15), we reasoned that simple growth assays with glucose would not be a reliable way to assess the ability of HPr-G54D to function in these phosphotransfer reactions.

**Fig 3:**
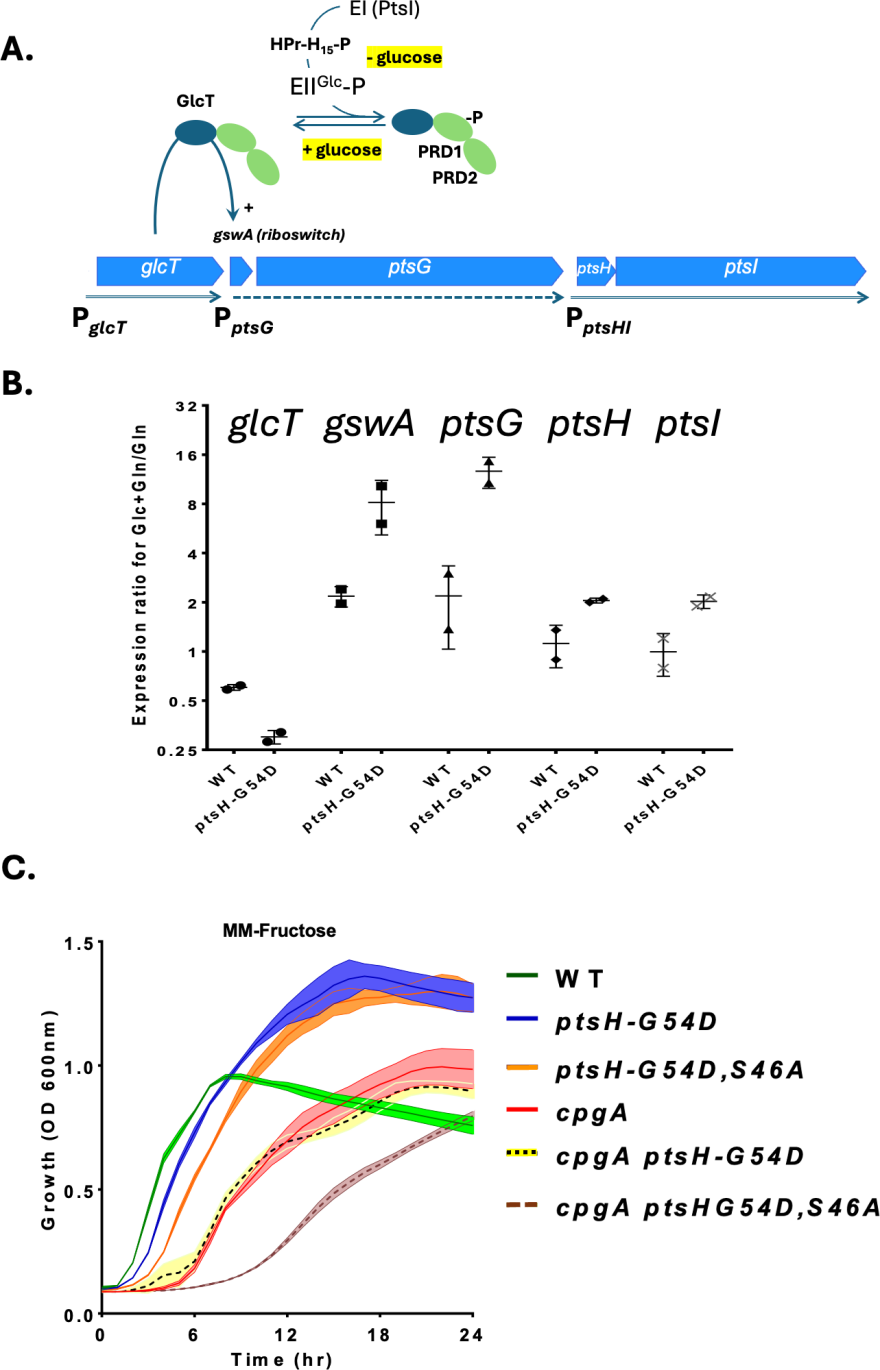
HPr-G54D still supports PTS-dependent sugar import. (**A**) Model for HPr-dependent regulation of *ptsG* (encoding EII^Glc^) by the GlcT antitermination protein. GlcT contains two PRDs and is active in its unmodified state. In cells grown in the absence of glucose, EI (PtsI) uses phosphoenolpyruvate to generate HPr-H15-P, which in turn donates a phosphoryl group to EII^Glc^. In the absence of glucose, the phosphorylated EII^Glc^-P transfers phosphate to the PRD1 of GlcT leading to inactivation of the antitermination protein and low expression of *ptsG*. If glucose is present, EII^Glc^-P is consumed by import of glucose (generating glucose-6-phosphate), GlcT remains in its unmodified, active state, and readthrough of the riboswitch (*gswA*) occurs, leading to elevated *ptsG* expression (dotted arrow). The neighboring *glcT* and *ptsHI* genes are each expressed from separate, constitutive promoters (solid arrows). (**B**) Quantitative RT-PCR was used to monitor the effect of glucose on mRNA levels for *glcT*, *gswA* (measuring readthrough *ptsG* riboswitch terminator), *ptsG*, *ptsH*, and *ptsI*. Strong antitermination of the *gwsA* riboswitch and increased *ptsG* transcription are observed in both the WT (168) and the *ptsH*-G54D (HBAS1854) strains in medium containing both gluconate (Gln) and glucose (Glc) compared to gluconate alone (Gln). (**C**) Growth was monitored in minimal medium (MM) supplemented with 1% fructose. The strains used were, in order, 168 (WT), HBAS1854, HBAS1835, HB20401, HBAS1855, and HBAS1838.

As an alternative approach to monitor the HPr-dependent phosphorylation of EII^Glc^, we used RT-PCR to monitor the GlcT-mediated, glucose-dependent induction of *ptsG* (Fig. 3A). The GlcT antiterminator protein is expressed from a constitutively active promoter and is encoded immediately upstream of the *ptsGHI* operon. The *ptsG* operon is regulated by *gswA* (16), an RNA antiterminator (RAT) element that overlaps a transcription terminator (17). In media lacking glucose, HPr-H15-P transfers phosphate to EII^Glc^, and this phosphate is then transferred to the first of two tandem PTS regulation domains (PRD1) in the GlcT antitermination protein (18). This inactivates GlcT, and *ptsG* transcription is terminated prior to the coding region (generating a truncated RNA annotated as S505). However, when glucose is present, EII^Glc^ transfers phosphate to glucose during import; GlcT is in its active, unphosphorylated form and binds to the RAT to allow readthrough into *ptsG*. This readthrough leads to elevated transcription of *ptsG* but has relatively little effect on the downstream *ptsH* and *ptsI* genes since these are independently transcribed from a strong promoter upstream of *ptsH* (Fig. 3A).

To monitor the activity of HPr and HPr-G54D in the reversible phosphotransfer from HPr-H15 to EII^Glc^, we used RT-PCR to compare gene expression in minimal medium (MM) + gluconate (non-inducing condition) with MM + gluconate + glucose (inducing condition). As expected for GlcT-mediated antitermination, readthrough of the terminator and expression of the downstream *ptsG* gene were induced ~4-fold when glucose was present (Fig. 3B). This induction was observed with both HPr and HPr-G54D, indicating that both are active in the H15-dependent phosphorylation of EII^Glc^, and when glucose is added, EII^Glc^-P supports glucose import, which leads to active (unphosphorylated) GlcT. The extent of glucose induction was even higher with the strain expressing HPr-G54D (Fig. 3B), which is the opposite of what might be expected if the mutant HPr was defective in phosphotransfer. As an independent measure of PTS-dependent sugar import, we monitored the effect of HPr-G54D on growth with fructose, a strictly PTS-dependent sugar. Growth on fructose was unimpaired relative to WT in the presence of the *ptsH*-G54D mutation (Fig. 3C). Together, these results argue against model 1, which proposed that HPr-G54D might be defective in support of glucose uptake. This could result from a decreased ability to be phosphorylated by enzyme I (PtsI) or a defect in phosphotransfer to EII^Glc^ (PtsG).

### Induction of GapA sensitizes ∆*cpgA* to gluconate

**Fig 4:**
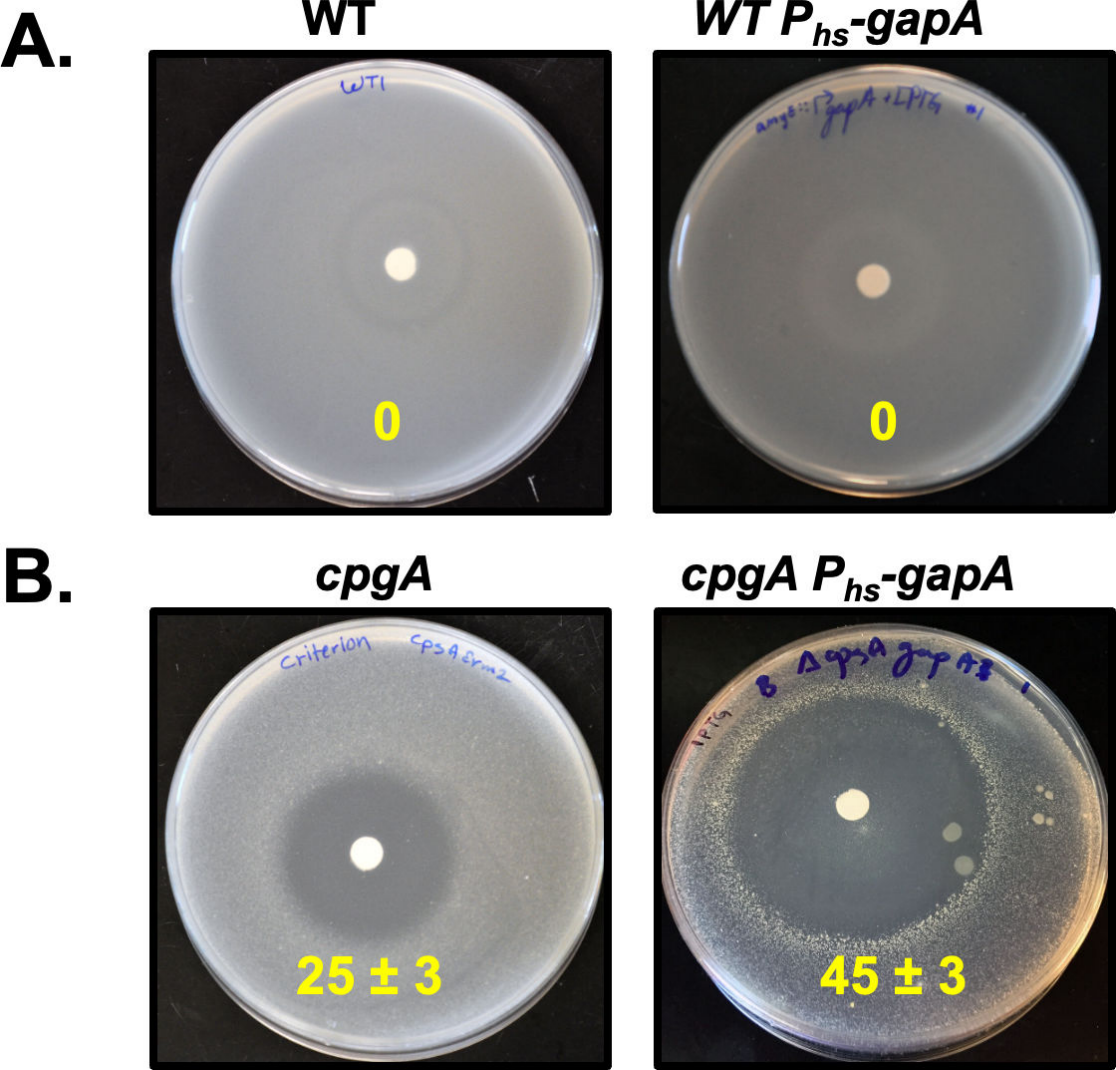
Induction of GapA sensitizes ∆*cpgA* to gluconate**.** Gluconate sensitivity was monitored by disk diffusion assay on MH media (zone of inhibition values are shown as diameter ± SEM, in mm). (**A**) No sensitivity to gluconate is seen in the WT strain with or without expression of an IPTG-inducible *gapA* (P_hs_-gapA) from an ectopic site (*amyE*) (HB21518). All P_hs_-constructs were tested in the presence of 0.5 mM IPTG. (**B**) In the ∆*cpgA* background (HB21527), expression of *gapA* increases gluconate sensitivity (from 25 to 45 mm). The suppressors that arise (colonies in the clear zone) contain mutations that eliminate or reduce expression of this ectopic copy. We used two-way ANOVA with multiple comparison along with Tukey’s post-corrections leading to the *P*-value of <0.001 for comparison of *cpgA*’s sensitivity to WT and ∆*cpgA amyE*::P_hs_-*gapA* (*n* = 3).

We next wished to test hypothesis 2, which proposes that (i) GapA is the protein that synthesizes 4PE in *B. subtilis* and (ii) HPr-G54D allosterically inhibits GapA to reduce the synthesis of toxic 4PE. Previous work suggests that 4PE synthesis results from a promiscuous reaction of GAPDH with E4P (6). In *B. subtilis*, the major GAPDH enzyme during growth on glycolytic substrates is GapA (19). Consistent with a role for GapA in the synthesis of 4PE, induction of *gapA* (*amyE*::P_hs_-*gapA*) greatly increased growth inhibition by gluconate in ∆*cpgA* cells (Fig. 4B) (from 25 ± 3 to 45 ± 3 mm), but not in WT cells where no gluconate-dependent toxicity was observed (Fig. 4A). These results support our premise that GapA is the protein that synthesizes 4PE in *B. subtilis*.

### The HPr-G54D substitution is found in other bacteria

**Fig 5:**
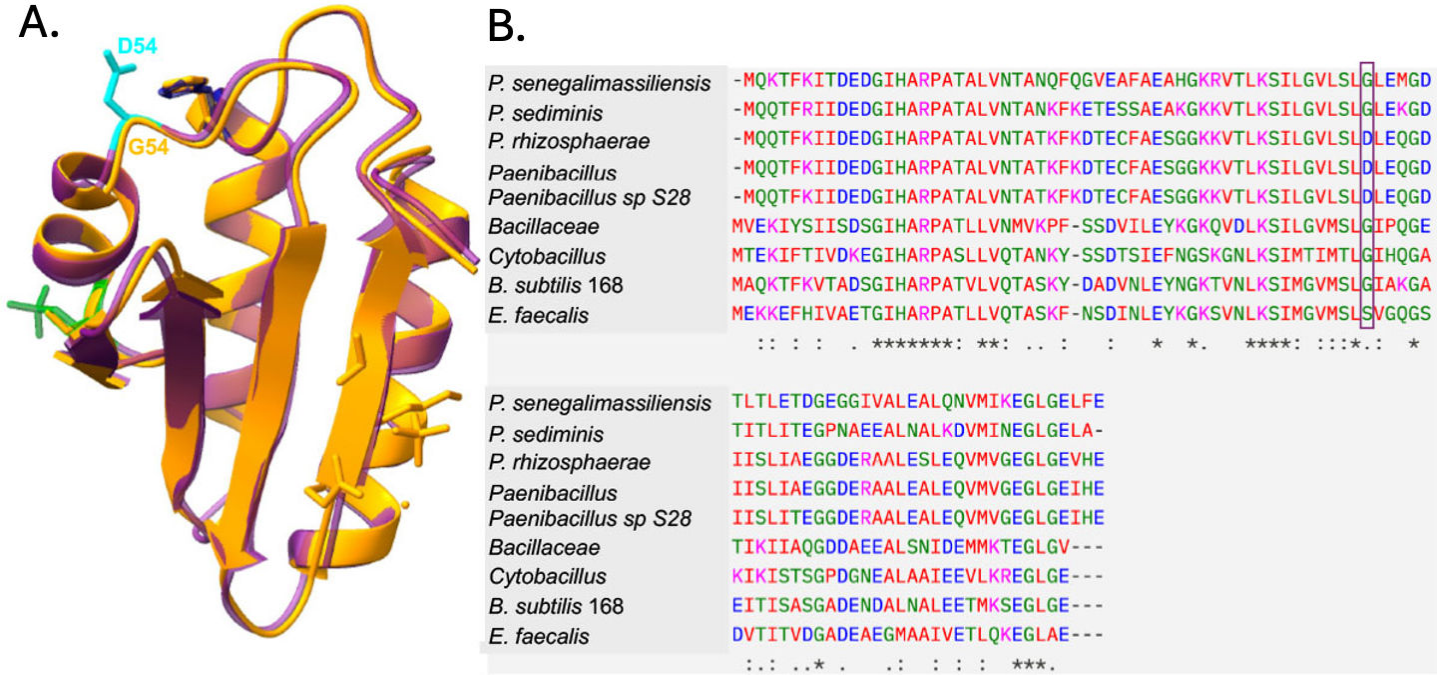
The HPr-G54D substitution is found in other bacteria. (**A**) The *B. subtilis* HPr structure (20) is shown in orange, with the predicted structure of HPr carrying S46-P (green) and D54 (cyan) overlayed in magenta using matchmaker command in ChimeraX. (**B**) Various HPr protein raw sequences were submitted to MUSCLE (EMBL server; [21]) in a FASTA format for generation of a multiple sequence alignment. The sequences shown are from top to bottom: various *Paenibacillus* spp. (*P. senegalimassiliensis* WP_059052857, *P. sediminis*, WP_209845805, *P. rhizosphaerae*, WP_183581586, *Paenibacillus* spp., WP_076169674, *Paenibacillus* sp. S28, WP_200631261), *Bacillaceae*, *Cytobacillus*, WP_209844287, *B. subtilis* 168, and *Enterococcus faecalis*, WP_251157054. The Gly54 position of *B. subtilis* 168 HPr and the aligned residues are boxed.

Inspection of the HPr structure reveals that the Asp54 residue (in G54D proteins) is near His15 (Fig. 5A) and on the same alpha helix as Ser46. The HPr G54D substitution affects a highly conserved Gly residue present in the vast majority of HPrs. However, a small number of HPrs from the *Paenibacillus* genus naturally have Asp at this position (Fig. 5B). In addition, the pathogen *Enterococcus faecalis* contains Ser at this position (Fig. 5B), which speculatively could provide a site for introduction of a negative charge by phosphorylation.

### HPr-G54D does not strongly impede GapA-dependent 4PE synthesis

HPr was previously found to bind to GapA by affinity tag co-purification (13). During growth on glucose, the majority of HPr is present in the HPr-S46-P form that is generated by the bifunctional HPrK (9, 10, 22). Prior biochemical evidence suggests that HPr-S46-P (at a 10:1 ratio), but not HPr alone or the HPr-H15-P form, inhibits GapA activity by up to 35% (13). This led us to hypothesize that the HPr-G54D protein might decrease GapA-dependent synthesis of toxic 4PE. Consistent with this hypothesis, a *ptsH*-G54D mutation partially rescued growth of a *cpgA* mutant on glucose plus gluconate, but a *cpgA ptsH*-S46A,G54D mutant did not (Fig. 2B). This suggests that growth rescue is likely mediated by the seryl-phosphorylated form of the mutant protein (HPr-S46-P, G54D).

To investigate how HPr affects *B. subtilis* GapA activity, we first assayed GapA with G3P as substrate (Fig, S3). GapA has a *K*_M_ for G3P (2.2 mM; Table 1), similar to that determined previously for the *Bacillus stearothermophilus* enzyme (0.9 mM) (23). Under our conditions, *k*_cat_ (481 s^−1^) was higher than reported previously (76 s^−1^), perhaps due to differences in the assay conditions or intrinsic differences in the protein preparations. Based on these values, we determined a catalytic efficiency (*k*_cat_/*K*_M_) of 219 mM^−1^ s^−1^ with G3P (Table 1). The catalytic efficiency of GapA with E4P was 200 times lower (*k*_cat_/*K*_M_ = 1.05 mM^−1^ s^−1^), consistent with the general expectation for a promiscuous enzyme reaction (24). Since we could not approach saturation with E4P (Fig. S3), we could not obtain reliable estimates for *K*_M_ and *k*_cat_, but we estimate that *K*_M_(E4P) was at least 10 mM under our conditions.

**TABLE 1:** Experimental calculation of *k*_cat_/*K*_M_ and estimated flux ratio for GapA

		GAPDH	GAPDH + HPr	GAPDH + HPr-G54D	GAPDH + HPr-S46E	GAPDH + HPr-G54D, S46E
G3P	*K*_M_ (mM)	2.2 ± 0.2	13.7 ± 0.2	6.2 ± 0.3	22.4 ± 1.2	4.0 ± 0.2
	*k*_cat_ (s^−1^)	481 ± 14	786 ± 71	423 ± 6	870 ± 28	657 ± 15
	*k*_cat_/*K*_M_ (mM^−1^ s^−1^)	219	57.4	68.5	38.8	164
E4P^*a*^	*k*_cat_/*K*_M_ (mM^−1^ s^−1^)	1.05	1.88	0.73	2.60	3.78
	G3P/E4P flux ratio (120 µM G3P, 1 mM E4P)	25	3.7	11	1.8	5.2

^*a*^ The catalytic efficiency (k_cat_/*K*_M_) was determined using linear regression of the reaction velocity vs substrate concentration plots assuming pseudo-first-order kinetics (where [S] < *K*_M_).

We next assayed GapA with G3P or E4P as substrates in the absence or presence of various purified HPrs (HPr, HPr-G54D, HPr-S46E, and HPr-S46E, G54D). Here, the S46E substitution is used to mimic the effect of a seryl-phosphate group, recognizing that phosphomimetic substitutions may not fully recapitulate the functional changes associated with phosphorylation (25). We hypothesized that HPr-G54D (*in vivo* in the HPr-S46-P,G54D form) might reduce 4PE synthesis either through a general inhibition of GapA activity or by increasing the ability of GapA to discriminate between the cognate substrate, G3P, and the non-cognate substrate E4P.

All of the HPr variants modestly reduced the catalytic efficiency (*k*_cat_/*K*_M_) of GapA with G3P, with the strongest effects noted for the HPr-S46E phosphomimetic protein (Table 1). These effects were almost entirely due to an increase in *K*_M_, suggestive of a reduced affinity for G3P. The increase in the GapA *K*_M_(G3P) was strongest with the HPr-S46E protein (~10-fold), and much weaker with the phosphomimetic HPr-S46E G54D protein (~2-fold). The effects on the much slower reaction with E4P were less uniform: HPr-G54D reduced catalytic efficiency activity relative to HPr alone, whereas both HPr-S46E and HPr-S46E G54D modestly increased catalytic efficiency. These effects of HPr on GapA activity are consistent with direct protein-protein interactions, and the conformation of various GapA:HPr complexes can be predicted using AlphaFold 3 (Fig. S4 and associated text).

We next estimated the *in vivo* flux through GapA with both substrates as described by Copley (24). This requires estimates for the *in vivo* concentrations of GapA, G3P, and E4P. GapA is abundant*,* with an estimated 19,200 copies per cell (~20 µM) in the presence of glucose (26). In cells, GapA likely functions with substrate levels near the low end of those measured here (Fig. S3). Aldolase cleaves fructose-1,6-bisphosphate into G3P and dihydroxyacetone phosphate (DHAP) (Fig. 1), which are rapidly equilibrated by triose phosphate isomerase. At equilibrium, the DHAP concentration is ~22 times greater than G3P, which we estimate as 120 µM (27). This is based on the equilibrium constant of the triose-phosphate isomerase (TPI) reaction and the observed concentration (2.5 mM) of DHAP in both yeast (27) and *B. subtilis* (28). This value is consistent with the measured concentration of G3P in yeast (27). In contrast, E4P is comparatively abundant, with levels of ~1 mM measured in *B. subtilis* (28). However, this is also well below *K*_M_(E4P), which we have estimated as >10 mM (Fig. S3). Thus, the concentrations of both GapA substrates (G3P and E4P) are an order of magnitude or more below their *K*_M_, implying pseudo-first-order kinetics.

We used these estimates for enzyme and substrate concentration to predict *in vivo* fluxes. The higher concentration of E4P (1 mM) relative to G3P (120 µM) partially compensates for the >200-lower catalytic efficiency, yielding a predicted flux ratio of ~25 in WT cells (96% of the GapA turnovers use G3P and 4% E4P). However, when we calculated the effect of HPr and HPr variant proteins on the predicted flux ratio, we noted lower values (flux ratios < 6). This change is due largely to the reduction of GapA *K*_M_(G3P) in the presence of HPrs (Table 1). The high frequency with which GapA appears to use E4P as substrate in place of G3P supports the idea of an underground metabolism (29).

We next sought to determine how HPrs might affect the synthesis of toxic 4PE in cells. Since both G3P and E4P are present in cells at levels well below their *K*_M_, the two substrates do not compete significantly for the active site. Therefore, the rate of synthesis of the toxic metabolite 4PE is determined primarily by the concentration of E4P and the catalytic efficiency of GapA (Table 1). The catalytic efficiency (*k*_cat_/*K*_M_) of GapA with HPr-G54D (0.73 mM^−1^ s^−1^) is ~2 times lower than for WT HPr (1.88 mM^−1^ s^−1^; Table 1), which could serve to reduce 4PE synthesis. However, the HPr-S46E, G54D mutant protein, here used as a surrogate for the presumed *in vivo* effector (HPr-S46-P,G54D), has the opposite effect (*k*_cat_/*K*_M_ = 3.78 mM^−1^ s^−1^). We conclude that increasing GapA is deleterious in *cpgA* mutant cells (Fig. 4), GapA can synthesize 4PE (Fig. S3), and HPr affects GapA activity *in vitro* (Table 1). However, the impact of HPr variants on GapA activity is not well correlated with the ability of HPr-G54D to increase fitness of the ∆*cpgA* strain. Thus, while we cannot rule out some contribution from allosteric regulation of GapA, these enzymology measurements do not provide support for the hypothesis that HPr-G54D functions by reducing 4PE synthesis (hypothesis 2).

### The HPr-G54D substitution alters CCR

**Fig 6:**
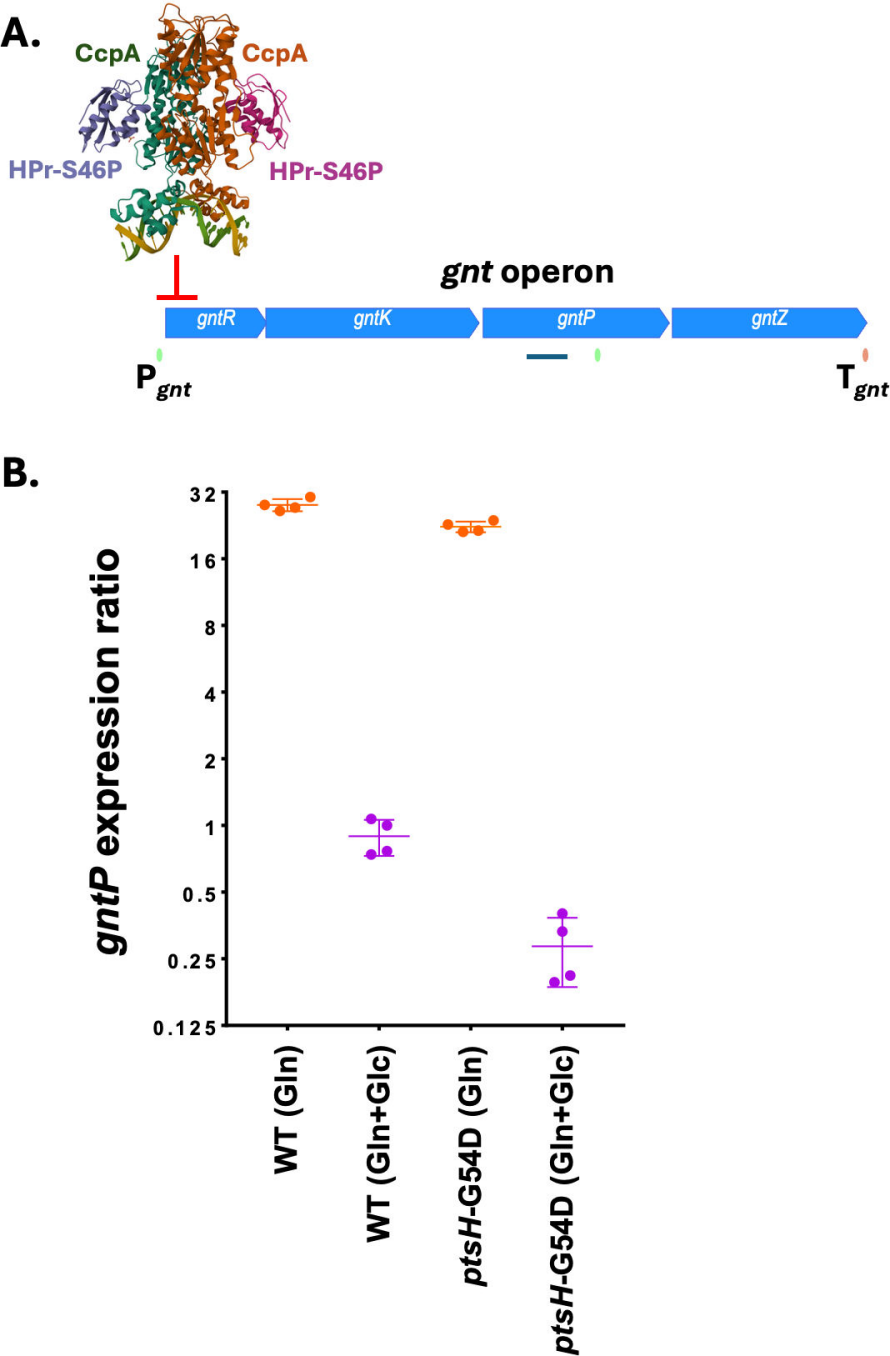
The HPr-G54D substitution alters CCR of the gluconate operon. (**A**) In the presence of glucose, HPr-S46-P binds the dimeric CcpA protein to repress expression of the gluconate operon (*gntRKP*Z), as visualized in the structure of the CcpA-HPr-S46-P complex bound to the *gntR* downstream *cre* site (PDB: 3OQN) (30). Transcription initiates from an upstream promoter (P*_gnt_*) and extends to the operon terminator (T*_gnt_*). (**B**) CCR was monitored by measuring the level of *gntP* RNA in WT (168) and *ptsH*-G54D (HBAS1854) strains in MH + gluconate (Gln) (non-repressing) compared to MH + Gln + glucose (Glc) (repressing) conditions. The RNA expression ratio for each condition is relative to *gntP* mRNA level in unamended MH medium. In MH + Gln, GntR-mediated repression is lost and the operon is induced. With Gln + Glc, CCR in the *ptsH*-G54D strain (0.28 ± 0.04) is significantly increased (*P* = 0.0007; unpaired, two-tailed *t*-test) relative to the WT strain (0.89 ± 0.08) (*n* = 4).

We next sought to test if the HPr-G54D protein increases CCR to help prevent the uptake of gluconate (hypothesis 3). In the presence of glucose, HPrK (HPr kinase/phosphorylase) is activated by fructose-1,6-bisphosphate and phosphorylates HPr on Ser46. HPr-S46-P then serves as a co-repressor with the dimeric CcpA DNA-binding protein to mediate CCR (Fig. 6A). Gluconate import and catabolism is controlled by the *gntRKPZ* operon (15), which is autoregulated by substrate induction mediated by the gluconate-sensitive repressor (GntR). In addition, the *gntRKPZ* operon is subject to CCR through binding of a complex of CcpA and HPr-S46-P (30).

We monitored the glucose-dependent CCR of the gluconate catabolism operon using RT-PCR to measure *gntP* expression (Fig. 6B). Consistent with prior results (31), this gene is well expressed in medium containing gluconate as inducer, but the addition of glucose leads to an ~32-fold decrease in *gntP* RNA. In the *ptsH*-G54D strain, CCR of *gntP* was at least three- to fourfold stronger than in the WT strain (Fig. 6B). Using AlphaFold 3, we predicted an increased interaction surface area (and ∆G of binding) between HPr-S46-P and CcpA as a result of the G54D substitution (Fig. S5). Thus, we suggest that the beneficial effect of the *ptsH*-G54D allele in the presence of gluconate is due, at least in part, to increased CCR of the *gntRKPZ* operon (Fig. 1; hypothesis 3). However, we cannot exclude the possibility that the *ptsH*-G54D allele may have additional effects on the other activities of HPr.

### The moonlighting function of CpgA is conserved among orthologs from pathogenic gram-positive bacteria

**Fig 7:**
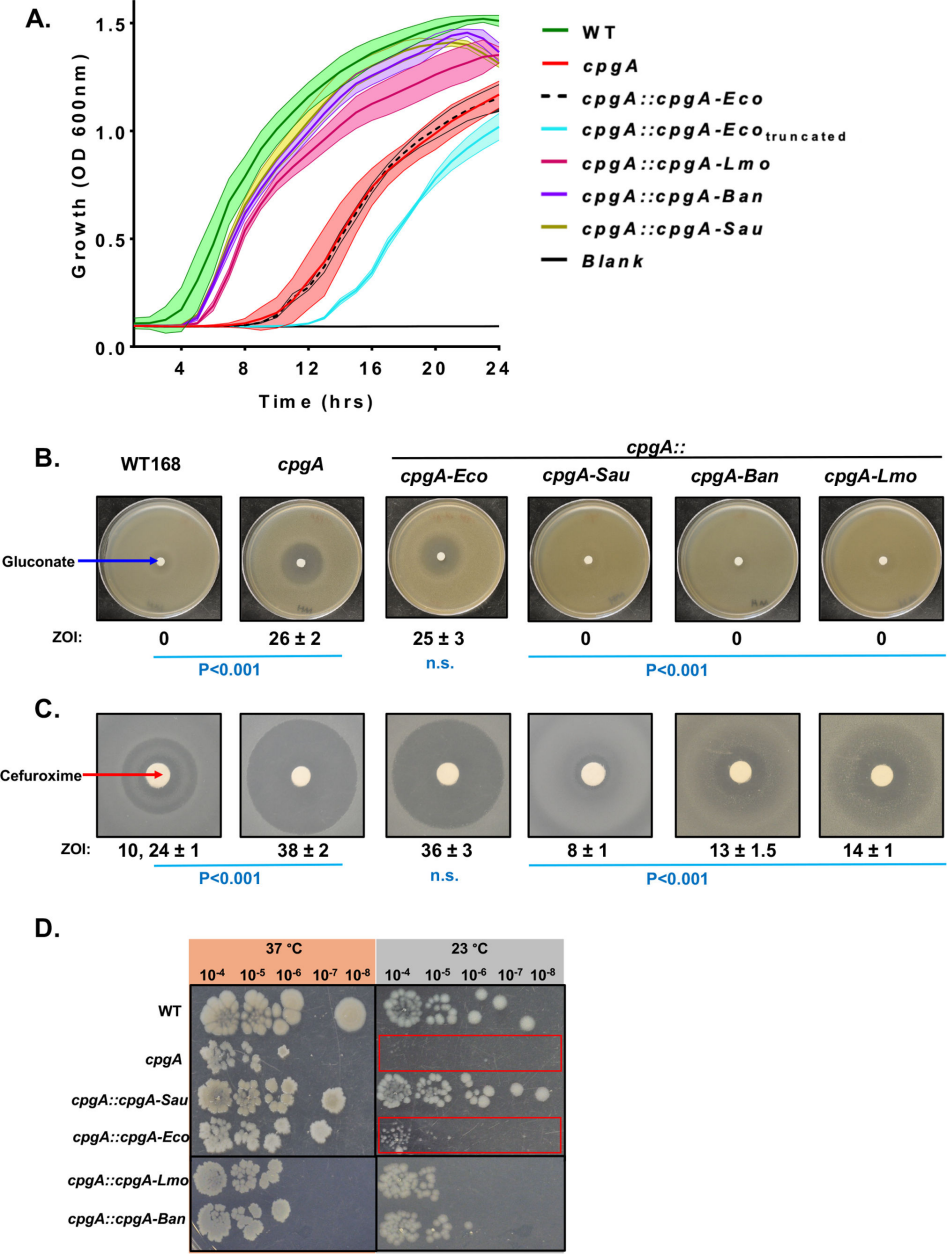
The moonlighting function of CpgA is conserved among orthologs from pathogenic gram-positive bacteria**.** (**A**) Aerobic growth of various strains monitored in LB broth aerobically at 37°C. The strains used were 168, HB20401, HBYL842, HBYL842.1, HBYL843, HBYL844, and HBYL845. (**B**) The images of gluconate disk diffusion assay on MH agar among various strains containing *cpgA* orthologs from other bacteria in place of *B. subtilis cpgA*. (**C**) The images of disk diffusion assay conducted on LB agar with cefuroxime disk. Two-way analysis of variance with multiple comparison along with Tukey’s *post hoc* correction was used for calculating *P*-value. All comparisons are shown with respect to *cpgA* null strain where *P* is <0.001 (*n* = 3) and n.s. represents no significant difference between sample types. (**D**) Aerobically grown cells were serially diluted, and 10 µL of culture was spotted onto LB agar. Identical plates were incubated at different temperatures. Spot dilution images for various strains grown on LB agar were captured. Red boxes highlight the weak rescue seen for growth at 23°C with the *E. coli* CpgA ortholog (RsgA) compared to ∆*cpgA* alone.

Our prior results reveal that CpgA plays an important role in metabolite proofreading by hydrolyzing 4-PE (5). Since GAPDH is a universally conserved protein, and production of 4-PE is a well-recognized promiscuous reaction (24, 32), we hypothesized that this proofreading role might be a conserved feature of CpgA orthologs. We selected orthologs from gram-positive bacteria based on both predicted protein homology and synteny. We additionally included the more distantly related ortholog from *E. coli*, RsgA (Fig. S6). To help ensure an appropriate expression level for each protein, we employed a CRISPR-based strategy to introduce *cpgA* orthologs at the native locus (33). The CpgA orthologs from *E. coli* (Eco), *Staphylococcus aureus* (Sau), *Listeria monocytogenes* (Lmo), *Bacillus anthracis* (Ban), and *E. coli* RsgA (Eco) were first tested for their ability to allow normal growth in LB medium, where ∆*cpgA* has a notable growth lag. With the notable exception of the *E. coli* ortholog (RsgA), the *cpgA* orthologs all complemented the ∆*cpgA* mutation (Fig. 7A). Alignment of the *E. coli* RsgA protein with CpgA (Fig. S7) revealed three non-aligned regions in RsgA. Therefore, we generated an *E. coli rsgA* allele lacking these non-aligned regions [Eco(trunc)], but expression of this version only further reduced fitness relative to the ∆*cpgA* strain (Fig. 7A).

The gram-positive CpgA orthologs also prevent metabolite intoxication during growth in the presence of gluconate (Fig. 7B). The ∆*cpgA* mutant has a significantly increased sensitivity to the second-generation cephalosporin CEF (Fig. 7C), a defect resulting from the dysregulation of central carbon metabolism (5). As also seen for gluconate sensitivity, the *S. aureus*, *B. anthracis*, and *L. monocytogenes* CpgA orthologs (but not *E. coli* RsgA) complemented the sensitivity of Δ*cpgA* cells to CEF (Fig. 7C). Thus, *E. coli* RsgA does not function to detoxify 4PE in *B. subtilis*, and consistently, an *E. coli rsgA::kan* strain was not sensitive to growth inhibition by glucose or gluconate (Fig. S8).

Since the *E. coli* RsgA protein was unable to complement the phenotypes resulting from metabolic dysregulation, we next sought to determine if this protein complements the ribosome assembly function of CpgA. Indeed, we observed weak complementation of the Δ*cpgA* cold-sensitive phenotype (Fig. 7D). Additionally, expression of *E. coli* RsgA complemented the modestly increased sensitivity of Δ*cpgA* cells to the protein synthesis inhibitors chloramphenicol and linezolid. This can be seen using eTest strips, where the relatively poor growth of ∆*cpgA* cells at 30°C is also visibly complemented (Fig. S9). These results suggest that CpgA orthologs from the Firmicutes retain the metabolite proofreading function, whereas *E. coli* RsgA functions only in ribosome assembly.

### *ptsH*-G54D suppresses antibiotic sensitivity of Δ*cpgA* cells

**Fig 8:**
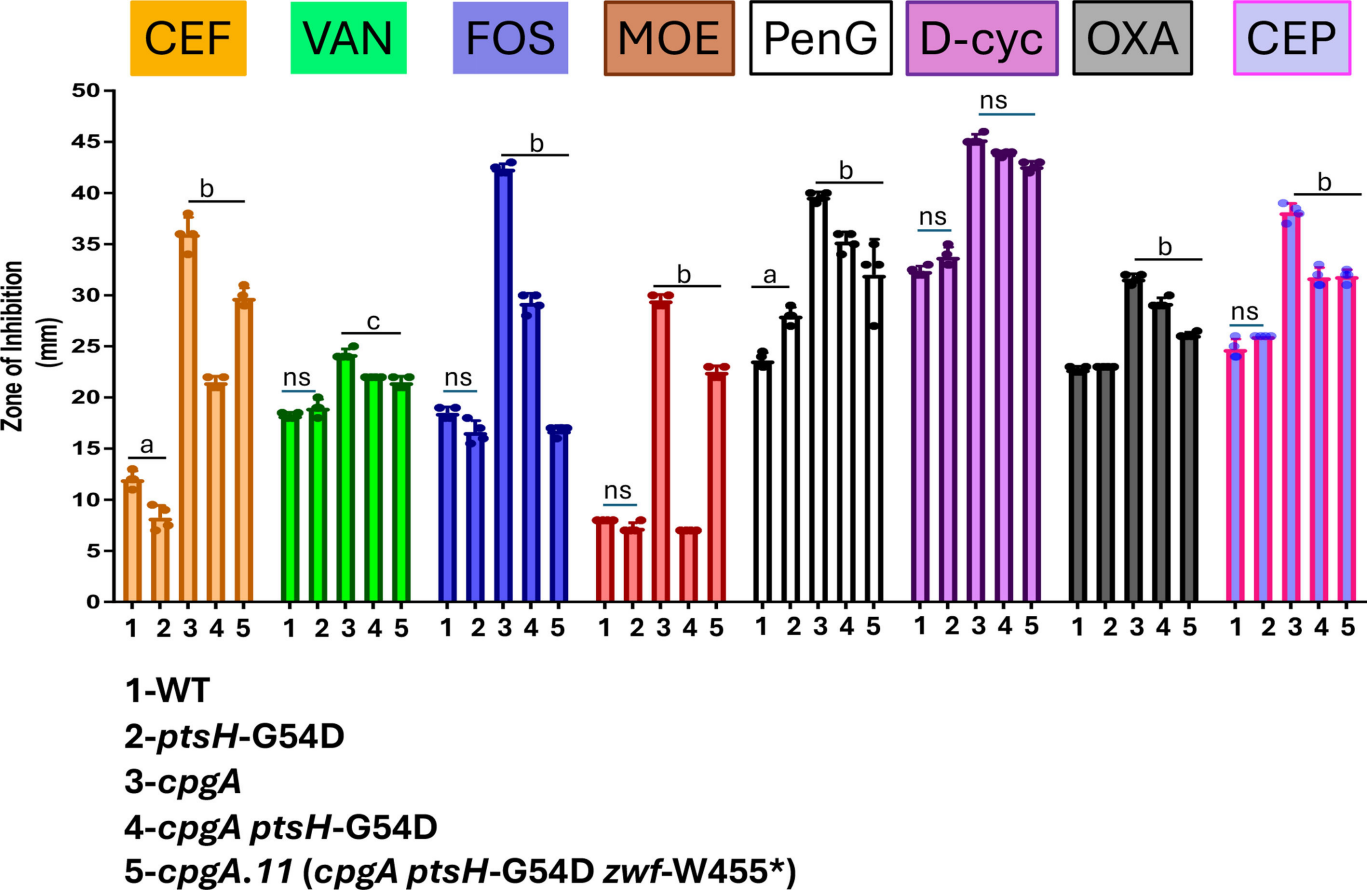
The *ptsH*-G54D allele provides resistance against inhibitors that target early stages of peptidoglycan assembly**.** The zone of sensitivity was measured against various cell wall inhibitors: 6 µg of cefuroxime disk, 10 µg of vancomycin, 100 µg of moenomycin disk, 1.5 µg of oxacillin, 3 µg of cephalexin, 60 µg of penicillin-G disk, 500 µg of fosfomycin disk, and 1.5 µg of D-cycloserine disk was conducted on LB agar. (*n* = 4). Two-way analysis of variance for multiple comparison was performed with Tukey’s post-correction. Here, letter a = comparison of between WT and *ptsH*-G54D strains (with *P* < 0.001), and b = comparison of *cpgA* to the other strains (with *P* < 0.001). c = comparison with ∆*cpgA* to that of the other strains with *P* < 0.01, whereas n.s. is used to denote no significant difference.

In the absence of CpgA, central carbon metabolism is dysregulated, and cells have an increased sensitivity to some peptidoglycan synthesis inhibitors (3, 5). To determine if the *ptsH*-G54D allele also suppresses antibiotic sensitivity in the Δ*cpgA* mutant, we tested a panel of antibiotics that impede peptidoglycan synthesis (Fig. 8). The ∆*cpgA* mutant was sensitive to the second-generation cephalosporin CEF and to the early-stage inhibitor fosfomycin, as previously reported (3, 5). Strong sensitivity was also noted for other β-lactam antibiotics and for the transglycosylase inhibitor moenomycin (Fig. 8).

The increased sensitivity to peptidoglycan synthesis inhibitors in the Δ*cpgA* strain was partially or completely suppressed by the *ptsH*-G54D allele (Fig. 8). In contrast, a *ptsH* null mutant, previously linked to resistance to the bacteriocin sublancin (34), was not beneficial. Our results further support the notion that antibiotic sensitivity in the ∆*cpgA* strain is a consequence of dysregulation of central metabolism ultimately leading to inhibition of Pgi (5). Prior work has shown that a loss of Pgi activity leads to a glucose-dependent accumulation of glucose-1-phosphate, inhibition of aminosugar synthesis, and ultimately cell lysis (35).

### Conclusion

We, here, provide evidence that *B. subtilis* GapA contributes directly to metabolite intoxication of ∆*cpgA* cells, consistent with the known ability of GAPDH enzymes to synthesize 4PE. We additionally show that the role of CpgA as a metabolite proofreading enzyme is conserved in several other gram-positive bacteria, and that in the absence of CpgA, metabolite intoxication sensitizes *B. subtilis* to a range of peptidoglycan synthesis inhibitors.

We previously isolated the *cpgA*.11 mutant by selecting for improved growth of a ∆*cpgA* strain in the presence of both glucose and gluconate. This suppressor carried two mutations: a truncation of *zwf* that reduces glucose-6-phosphate dehydrogenase activity to restrict the flux of glucose-6-phosphate into the PPP and a missense mutation, *ptsH*-G54D, encoding HPr-G454D. HPr, a key regulator and hub protein, is essential for the import of PTS sugars, a cofactor (with CcpA) for CCR, part of the sensory cascade that controls the activity of PRD-containing regulatory proteins, and an allosteric regulator of GapA. Here, we tested several hypotheses for the mechanism of action of the altered function HPr G54D protein. We conclude that phenotypic suppression most likely results from increased CCR of the gluconate catabolism operon, and this mutant protein is still competent for the import of PTS sugars, the regulation of the PRD-containing GlcT anti-termination protein, and allosteric interactions with GapA. Thus, in the original suppressor strain (cpgA.11), the *zwf* mutation functions to restrict flux of glucose into the PPP, and *ptsH*-G54D restrict gluconate import and catabolism. These results provide an example of how hub proteins can evolve mutations that selectively affect interaction with some, but not all, of their interaction partners in ways that benefit cell physiology.

## MATERIALS AND METHODS

### Bacterial strains and growth conditions

All primers (Table S1) and bacterial strains (Table S2) used in this study are listed. Bacterial strains were streaked onto LB agar medium from frozen glycerol stocks and were grown at 37°C overnight. Single isolated clones were picked for inoculation into MH or LB and were grown until mid-log phase (OD_600_ ~0.4–0.6). Such cultures were used as a source of inoculum (50-fold dilution in final volume) for either determining growth in MH or minimal inhibitory concentration (MIC) of various antibiotics in LB broths.

Chemically defined MM contains 10 g/L ammonium sulfate (NH_4_)_2_SO_4_, 5 g/L trisodium citrate (Na_3_C_6_H_5_O_7_.2H_2_O), 5 g/L L-glutamic acid (potassium salt monohydrate), 40 mM 3-(N-morpholino)propanesulfonic acid (MOPS) buffer (pH 7.4 using KOH), 2 mM KPO_4_ (pH 7.0), 10 mg/L tryptophan, 0.8 mM MgSO_4_, 15 µM ferric ammonium citrate, and 80 nM MnCl_2_. Carbon sources were added as follows: 0.5% glucose + 0.5% gluconate (MM-glucose + gluconate), or other indicated carbon sources. Mueller-Hinton broth (Hardy Diagnostics CRITERION) contains acid hydrolysate of casein 17.5 g/L, beef extract 2.0 g/L, and starch 1.5 g/L with a final pH of 7.3 ± 0.1 at 25°C. Where indicated, growth assays were performed in the presence of varying concentrations of kanamycin (0 μg–6 μg), tetracycline (0 μg–3 μg), or erythromycin (0 ng–500 ng). Growth measurements were made under shaking conditions monitored at 37°C using a BioTek H1 Synergy plate reader.

### CRISPR-based gene editing for heterologous expression

CRISPR-based gene editing was used to replace the *B. subtilis cpgA* gene at locus with *S. aureus* (N315), *B. anthracis* (Ames), *L. monocytogenes* (EGD-e), and *E. coli* (DH5α) homologs as described (33). Briefly, *cpgA* homologs were amplified and were fused to upstream and downstream fragments (~700 bp) relative to *B. subtilis cpgA* using long flanking homology PCR. This repair template (with Sfil-recognition sequences at the 5´ and 3´ end) was restriction digested and cloned into pAJS23 (33), which encodes a guide RNA targeted to the *erm* gene present in the Bacillus knockout erythromycin resistant (BKE) collection of *B. subtilis* strains (36). The resulting plasmids were transformed into *E. coli* DH5α cells followed by maintenance in *E. coli* TG1 for generating concatemeric DNA. Purified concatemeric plasmid DNAs were transformed into *B. subtilis cpgA::erm* knockout strains at permissive temperatures (30°C) and selected for kanamycin (15 µg mL^−1^) resistance on LB with 0.2% mannose. After 48 h, clones were patched on LB plates at a non-permissive temperature (45°C) for several generations. Colonies were tested for the loss of kanamycin and erythromycin resistance followed by verification with PCR and Sanger sequencing.

### Disk diffusion assay

Disk diffusion assays were performed as described previously in reference 5. In brief, 5 mL of bacterial cultures were grown in LB to mid-exponential phase (OD_600_ ~0.4) and were used as an inoculum into 5 mL of top, soft MH (for gluconate) or soft LB (for antibiotics) agar containing 0.75% agar held at 50°C. These cultures in top agar were mixed and gently poured onto 15 mL of bottom, hard MH or LB agar (final 1.5% agar), and were allowed to solidify for 30 min. To measure sensitivity, 6 µL of cefuroxime (1 µg mL^−1^), 5 µL of vancomycin (2 µg mL^−1^), 4 µL of moenomycin (25 µg mL^−1^), 3 µL of oxacillin (0.5 µg mL^−1^), 2 µL of cephalexin (1.5 µg mL^−1^), 4 µL of penicillin-G (15 µg mL^−1^), 10 µL of fosfomycin (50 µg mL^−1^), 15 µL of D-cycloserine (100 µg mL^−1^), or 16 µL of 25% gluconate was impregnated onto 8 mm Whatman filter paper disks. These plates were incubated at 37°C for 18 h, and the diameter of the zone of clearance was measured around the disks. In some cases, the zone of clearance was surrounded by a second zone with low-density growth.

### Chloramphenicol and linezolid MIC determination

Cells were aerobically grown in LB broth until OD_600_ ~0.4, and 100 µL was transferred to 5 mL of LB soft agar, which was mixed and overlayed onto LB hard agar (15 mL) and solidified at room temperature. The chloramphenicol and linezolid MIC strips (Liofilchem) were placed, and the plates were incubated at 30°C.

### Colony size measurement

Colony size was calculated as described in reference 5. Briefly, overnight cells grown from LB agar were grown in liquid suspension and were grown to mid-log phase, followed by 100 µL of culture from 10^−5^ serial dilutions being dispensed onto LB agar (20 mL each wt/vol with or without 0.5 mM IPTG). These plates were incubated to get well-separated, countable single clones which were imaged using Canon EOS 90D DSLR camera. The captured images were processed using Fiji-ImageJ software to determine the area of individual clones.

### Expression and purification of His-tagged GapA and HPr and HPr variants

*B. subtilis* GapA and HPrs were purified as His-tagged proteins after expression in *E. coli*. DNA fragments encoding *B. subtilis ptsH*, *ptsH* G54D (GGT to GAT), *ptsH* S46E (TCT to GAA), and *ptsH* G54D S46E were commercially synthesized and cloned into pMCSG19c vector, which allows for the T7 RNA polymerase-driven expression of His-tagged HPr as a maltose binding protein-tobacco vein mottling virus (TVMV)-His-HPr fusion and *in vivo* cleavage of the maltose-binding protein (MBP) domain using the TVMV protease (37). The resultant constructs were transformed into *E. coli* BL21(DE3) pLysS. *B. subtilis* GapA was purified from a previously described GapA-pWH844 plasmid in *E. coli* DH5α (38).

Cells were grown in 1 L of LB broth with 100 µg/mL ampicillin at 37°C with shaking to an OD_600_ of 0.4. IPTG was added to 1 mM final concentration, and the cultures were incubated at room temperature (~20°C) with shaking overnight (~14 h). Cells were collected by centrifugation, and the His-tagged proteins were purified using Ni-affinity columns.

For GapA purification, cells were resuspended in Ni-NTA buffer (50 mM NaH_2_PO_4_ pH 8, 300 mM NaCl, 10 mM imidazole, 10 mM DTT, and 5% glycerol) containing an EDTA-free protease inhibitor cocktail (Pierce Cat # A32965). Cells were lysed using French Press, followed by sonication, and cell debris was removed by centrifugation at 18,000 × *g* for 10 min. HisPur Ni-NTA Superflow agarose (Thermo Scientific Cat # 25214) column was prepared by packing a 10 mL resuspended resin (~5 mL packed resin) into a 25 mL empty gravity column. The resin was washed with 10-column volumes of Ni-NTA buffer with 10 mM imidazole. The clarified lysate was manually loaded on the HisPur Ni-NTA Superflow agarose column, and the resin was washed thrice with five-column volumes of Ni-NTA buffer with 20, 30, and 50 mM imidazole. His-GapA was eluted using a 100 to 300 mM imidazole gradient in 1 mL fractions. The different fractions were tested using the Bradford assay and SDS-PAGE. GapA-containing fractions were dialyzed overnight against a 50 mM Tris-HCl, pH 8, 150 mM NaCl, 10 mM DTT, and 10% glycerol.

For HPr purification, cells were resuspended in Ni-NTA buffer (50 mM NaH_2_PO_4_ pH 8, 300 mM NaCl, 10 mM imidazole, and 5% glycerol) containing an EDTA-free protease inhibitor cocktail (Pierce Cat # A32965). Cells were processed as described above, and the His-HPrs were purified using a fast protein liquid chromatography (FPLC) system (Pharmacia). The clarified lysate was applied to a 5 mL HisTrap FPLC column at a 1 mL/min flow rate, and proteins were eluted using a 1 h 10 to 300 mM imidazole gradient. HPr-containing fractions were dialyzed overnight against a 50 mM Tris-HCl, pH 8, 150 mM NaCl, and 10% glycerol. Purified proteins were quantified spectrophotometrically at 280 nm using their specific protein extinction coefficient values.

### GAPDH enzymatic activity

GAPDH activity was measured using arsenolysis reactions as described (39). Briefly, buffer exchange of GapA protein was performed using a Micro Bio-Spin P-6 Gel column (BioRad Cat # 7326221) equilibrated in 50 mM Tris-HCl, pH 8, 150 mM NaCl, and 10% glycerol buffer. Buffer-exchanged GapA (2.5 mM) was equilibrated for 15 min in reaction buffer (125 mM triethanolamine, pH 8, 5 mM L-cysteine, and 20 mM potassium arsenate), and the reactions were initiated by the addition of 2.5 mM NAD and different concentrations of D-glyceraldehyde-3-phosphate (Sigma Cat #39705) or D-erythrose-4-phosphate (Sigma Cat #E0377). GAPDH activity was monitored by following the level of NAD reduction spectrophotometrically at 340 nm with 6 s intervals for 10 min using an H1 Synergy plate reader (BioTek Instruments, Inc., VT). The amount of NADH produced between the 60 s to 120 s intervals was used to calculate the initial velocity of the reaction (39). Enzymatic reaction parameters (*k*_cat_, *K*_M_) were estimated using the Michaelis-Menten enzyme kinetics nonlinear regression method in GraphPad Prism 8 with the default settings.

### Real-time RT-PCR

RNA was extracted from cells aerobically grown in MH broth to mid-log phase and then treated with and without different carbon sources (0.1% gluconate or 0.1% each of glucose plus gluconate) for 30 min with shaking. Cells were harvested, lysed using lysozyme, and subjected to RNA extraction and purification as per the instructions of Qiagen RNeasy RNA extraction kit followed by DNase treatment (Ambion). RNA with the highest purity was used for complementary DNA (cDNA) synthesis, where 1 ug of total RNA from each condition was used to generate cDNA using Applied Biosystems High-Capacity cDNA reverse transcription (Thermo) kit. Furthermore, 10 ng of cDNA for *gntP* expression and 30 ng of cDNA for *glcT-gswA-ptsGHI* operon were used as an input cDNA for gene expression analysis tested using Applied Biosystems Power Up SYBR Green Master Mix (Thermo) in QuantStudio3 (Thermo). The *gyrA* gene was used as an internal reference for normalizing expression across samples. RNA was isolated from multiple independent cultures from distinct colonies for *gntP* (*n* = 4) and the *glcT* and *pts* operon genes (*n* = 3).

### AlphaFold-multimer modeling

The AlphaFold server (40) was used to predict the structure and interaction of HPr with GapA or CcpA. Since GAPDH is a tetrameric protein, AlphaFold-multimer modeling was done using four GapA and four HPr monomers. The predicted protein structures were viewed and edited in the UCSF ChimeraX tool (41), and the interface area and free energies from individual subunit-subunit interactions were calculated using the PDBe Protein Interfaces, Surfaces and Assemblies server.

## SUPPLEMENTAL MATERIAL

Supplemental Material jb.00162-25-s0001.pdf Tables S1 to S3; Fig. S1 to S9.

ASM does not own the copyrights to Supplemental Material that may be linked to, or accessed through, an article. The authors have granted ASM a non-exclusive, world-wide license to publish the Supplemental Material files. Please contact the corresponding author directly for reuse.
