# Supplementary material for: A ptsH mutation suppresses growth defects and antibiotic sensitivity in a cpgA mutant defective in metabolite proofreading
Source: J Bacteriol. 2025 Aug 14;207(9):e00162-25. doi: 10.1128/jb.00162-25 (PMC12445079; doi:10.1128/jb.00162-25)
Supplement: Supplemental Material — Tables S1 to S3; Fig. S1 to S9. [file jb.00162-25-s0001.pdf]

Supplementary Information (SI)

A *ptsH* mutation suppresses growth defects and antibiotic sensitivity in a *cpgA* mutant defective in metabolite proofreading

Ankita J. Sachla, Ahmed Gaballa, Diana Herrera, John D. Helmann[†]

List of SI materials

SI Table 1: Primers used in this study
SI Table 2: Strains used in this study
SI Table 3. Modeling of the HPr-GapA interaction using AlphaFold 3

Fig S1: Colony size for various strains
Fig S2: The *ptsH*-G54D mutation is unable to suppress cold sensitivity of Δ*cpgA*
Fig S3: Kinetics of GapA (GAPDH) with G3P and E4P
Fig S4: Phosphorylation of HPr S46 and G54D substitution alter the HPr interaction with GapA.
Fig S5: HPr G54D increases the HPr interaction interface with CcpA
Fig S6: Maximum likelihood (ML) phylogeny inferred from CpgA family protein multiple sequence alignment.
Fig S7: Multiple sequence alignment of CpgA orthologs
Fig S8: *E. coli rsgA*::*kan* is not sensitive to glucose or gluconate
Fig S9: Chloramphenicol and linezolid MIC determination by antibiotic strips.

SI Table 1: Primers used in this study.

Primer Name	Sequence (5'-3')
pdxBFXbaI	CAGTTCTAGAAAAGGAGGAAGGATCAGTGAAAATCCTTGTTGATG
pdxBRBglII	ATATAGATCTTTAACGTGCCGGATGATGAAC
prkCFrepair	AAGGCCAACGAGGCCACGGATCCTAAAGCGGATACCACAG
rpe-downrepair-R	AAGGCCTTATTGGCCTAGATCGGGAATGAGATTTTTCGGGCCTC
LFHprkCrsgAR	CTTTGGAGAGTTTATTTTTACTCAAATTTTCCCTCCTTGTTATTCATCTTTC
LFHprkCrsgAF	GAAAGATGAATAACAAGGAGGGAAAATTTGAGTAAAAATAAACTCTCCAAAG
LFHrsgArpeF	GTAAAAACTTTTCTGATACGGATGACTGACATAATGATAAAGGTTGCACCATCTATTC
LFHrsgArpeR	GAATAGATGGTGCAACCTTTATCATTATGTCAGTCATCCGTATCAGAAAAGTTTTTAC
LFHprkCstaphR	GATTTCACTATTCGACCTGTCTTCAAATTTTCCCTCCTTGTTATTCATCTTTC
LFHprkCstaphF	GAAAGATGAATAACAAGGAGGGAAAATTTGAAGACAGGTCGAATAGTGAAATC
LFHstaphrpeF	GAAATTTCAAATAGAAAGGTTAGATATTAACATAATGATAAAGGTTGCACCATCTATTC
LFHstaphrpeR	GAATAGATGGTGCAACCTTTATCATTATGTTAATATCTAACCTTTCTATTTGAAATTTC
LFHprkClmoAR	CAACGCTTTGATAATTTGTCCTTCCAGCATATTTTCCCTCCTTGTTATTCATCTTTC
LFHprkClmoF	GAAAGATGAATAACAAGGAGGGAAAATATGCTGGAAGGACAAATTATCAAAGCGTTG
LFHlmorpeF	GAATTAAAAAACAGAAAGCCGAGGTATTAACATAATGATAAAGGTTGCACCATCTATTC
LFHlmorpeR	GAATAGATGGTGCAACCTTTATCATTATGTTAATACCTCGGCTTTCTGTTTTTTAATTC
LFHprkCBanAR	CTTTGGAGAGTTTATTTTTACTCAAATTTTCCCTCCTTGTTATTCATCTTTC
LFHprkCBanF	GAAAGATGAATAACAAGGAGGGAAAATATGCCAGAAGGAAAAATTGTAAAAGCTCTAAG
LFHBanrpeF	GAAATTAGAGAGAGAAAGCCGAGGTATTAGCATAATGATAAAGGTTGCACCATCTATTC
LFHBanrpeR	GAATAGATGGTGCAACCTTTATCATTATGCTAATACCTCGGCTTTCTCTCTCTAATTTC
ptsHF-SalI	ACACGTCGACATGGCACAAAAAACATTTAAAGTAACTG
ptsHR-HindIII	ACACAAGCTTTTACTCGCCGAGTCCTTCGCTTTTC
RT-PCR primers	
glcT-F	GCGATCAAAAGGCAGCAGCAGGGATTTG
glcT-R	CAGCGGACGGTTTGTCAACGCTGAAT
gswA-F	ACGGGAAGTTCATTCTCGTTCTTTTGCGC
gswA-R	TCATGACAACGAATTCACAGGCGTAAGTCTTCCAG
ptsG-F	TTTCGCAGTAGGTGTAGCCATC
ptsG-R	TCAACTGAATCCGAAGGAATG
ptsH-F	TGCAGATTCTGGAATCCATG
ptsH-R	TCGTCAGCTCCGGAAGC
ptsI-F	TAGTTCTCAGTGACCCTGAGCTTC
ptsI-R	TTACATCGCGGATATCTGCCGCAC
gntP-F	TATTTCCCTGCTGGTCGTATCGTTC
gntP-R	AATCCGCGATCAGTTTGCCCAGCAT
gyrA-F	GGCGGCCATGCGTTATACAG
gyrA-R	GCCATACCTACCGCAATGCC

SI Table 2: Strains used in this study.

Strain	Genotype	Construction	Reference
B. subtilis			
168	*trpC2*	Lab strain	Lab stock
HB20401	*trpC2 cpgA*::*erm*	BGSC→168	{Sachla, 2019 #5
HB20471	*trpC2 ΔcpgA ptsH::erm*	BKE→HB20409 (1)	This study
HB20686	*trpC2 ΔcpgA*::*cpgA-pMUTIN4* (*erm*)	*cpgA*-pMUTIN4-HB20409	(1)
HBYL842	*trpC2 cpgA*::*cpgA-Eco*	CRISPR→HB20401	This study
HBYL842.1	*trpC2 cpgA*::*cpgA-Eco(trunc)*	CRISPR→HB20401	This study
HBYL843	*trpC2 cpgA*::*cpgA-Lmo*	CRISPR→HB20401	This study
HBYL844	*trpC2 cpgA*::*cpgA-Sau*	CRISPR→HB20401	This study
HBYL841	*trpC2 cpgA*::*cpgA-Ban*	CRISPR→HB20401	This study
HB21518	*trpC2 amyE::P_{hs}-gapA (cat)*	pPL82-*gapA*→168	This study
HB21527	*trpC2 cpgA::erm amyE::P_{hs}-gapA (cat)*	pPL82-*gapA*→HB20401	This study
HBAS1854	*trpC2 ptsH-G54D*	CRISPR→168	This study
Δ*cpgA*.11	*trpC2 cpgA*::*erm ptsH-G54D zwfTrp455**	Suppressor of HB20401	(1)
HBAS1855	*trpC2 cpgA*::*erm ptsH-G54D*	CRISPR→HB20401	This study
HBDH1	*trpC2 cpgA::erm amyE::P_{hs}-ptsH (cat)*	pPL82-*ptsH*→HB20401	This study
HB24765	*trpC2 cpgA::erm amyE::P_{hs}-ptsH-G54D (cat)*	pPL82-*ptsH*-G54D→HB20401	This study
HB24167	*trpC2 cpgA*::*erm amyE::P_{hs}-pdxB*	pPL82-pdxB→HB20401	This study
HB21549	*trpC2 cpgA*::*erm amyE::P_{hs}-pgi*		(1)
HBAS1835	*trpC2 ptsH-S46A,G54D (cat)*	gblock→168	This study
HBAS1838	*trpC2 cpgA::erm ptsH-S46A,G54D (cat)*	*cpgA*::*erm* gDNA→HBAS1835	This study
E. coli			
pGP704	*pWH844-N-His-gapA* in DH5α		(2)
BL21(DE3)pLysS	F–, *omp*T, *hsd*S_B (r_B–, m_B–), *dcm*, *gal*, λ(DE3), pLysS, Cm^r	Lab strain	Lab stock
HE31401	*E. coli* BL21(DE3) pLysS pMCSG19c-*ptsH*	gblock of *ptsH* ORF cloned in pMCSG19c plasmid under IPTG induction	This study
HE31402	*E. coli* BL21(DE3) pLysS pMCSG19c-*ptsH* G54D	gblock of *ptsH*-G54D ORF cloned in pMCSG19c plasmid under IPTG induction	This study

HE31403	*E. coli* BL21(DE3) pLysS pMCSG19c-*ptsH* S46E	gblock of *ptsH*-S46E ORF cloned in pMCSG19c plasmid under IPTG induction	This study
HE31404	*E. coli* BL21(DE3) pLysS pMCSG19c-*ptsH* G54D S46E	gblock of *ptsH*-S46A, G54D ORF cloned in pMCSG19c plasmid under IPTG induction	This study
Plasmids			
pMCSG19c	Expression of MBP fusion and *in vivo* protein cleavage		(3)

SI Table 3. **Modeling of the HPr-GapA interaction using AlphaFold3.**

	ipTM	pTM	Interface area by GapA subunit / ΔG			Total interface area
GapA-HPr	0.83	0.86	370.6 Å^2 -5.1 kcal/mol	237.1 Å^2 0.7 kcal/mol	207.8 Å^2 -0.1 kcal/mol	815.5 Å^2
GapA-HPr-S46-P	0.71	0.76	460 Å^2 -7 kcal/mol	435.5 Å^2 -5.9 kcal/mol	249.2 Å^2 -0.1 kcal/mol	1144.7 Å^2
GapA-HPr-S46E	0.75	0.79	158.2 Å^2 -1.3 kcal/mol	607.2 Å^2 -1.5 kcal/mol	313.5 Å^2 -4.6 kcal/mol	1078.9 Å^2
GapA-HPr-G54D	0.8	0.83	438.2 Å^2 -5.5 kcal/mol	159.9 Å^2 -0.7 kcal/mol	229.1 Å^2 -0.9 kcal/mol	827.2 Å^2
GapA-HPr-S46-P,G54D	0.7	0.75	370.4 Å^2 -6.1 kcal/mol	370.4 Å^2 -6.1 kcal/mol	197 Å^2 0.1 kcal/mol	937.8 Å^2
GapA-HPr-S46E,G54D	0.77	0.81	455.1 Å^2 -7 kcal/mol	113.6 Å^2 -1.2 kcal/mol	237.7 Å^2 -2 kcal/mol	806.4 Å^2

AlphaFold 3 (4) was used to predict the structure and interaction of GapA and different forms of HPr. The prediction confidence metrics scores are listed as predicted template modeling (pTM) and the interface template modeling (ipTM) scores. The predicted structures were assessed by calculating the interface area and free energies from individual subunit-subunit interactions using the PDBe Protein Interfaces, Surfaces, and Assemblies (PDBe PISA) server (5). The interface area and free energies of the interaction between an HPr monomer and GapA monomers (yellow, pink, and dark Salmon in Fig S5) are listed in columns 4, 5, and 6, respectively.

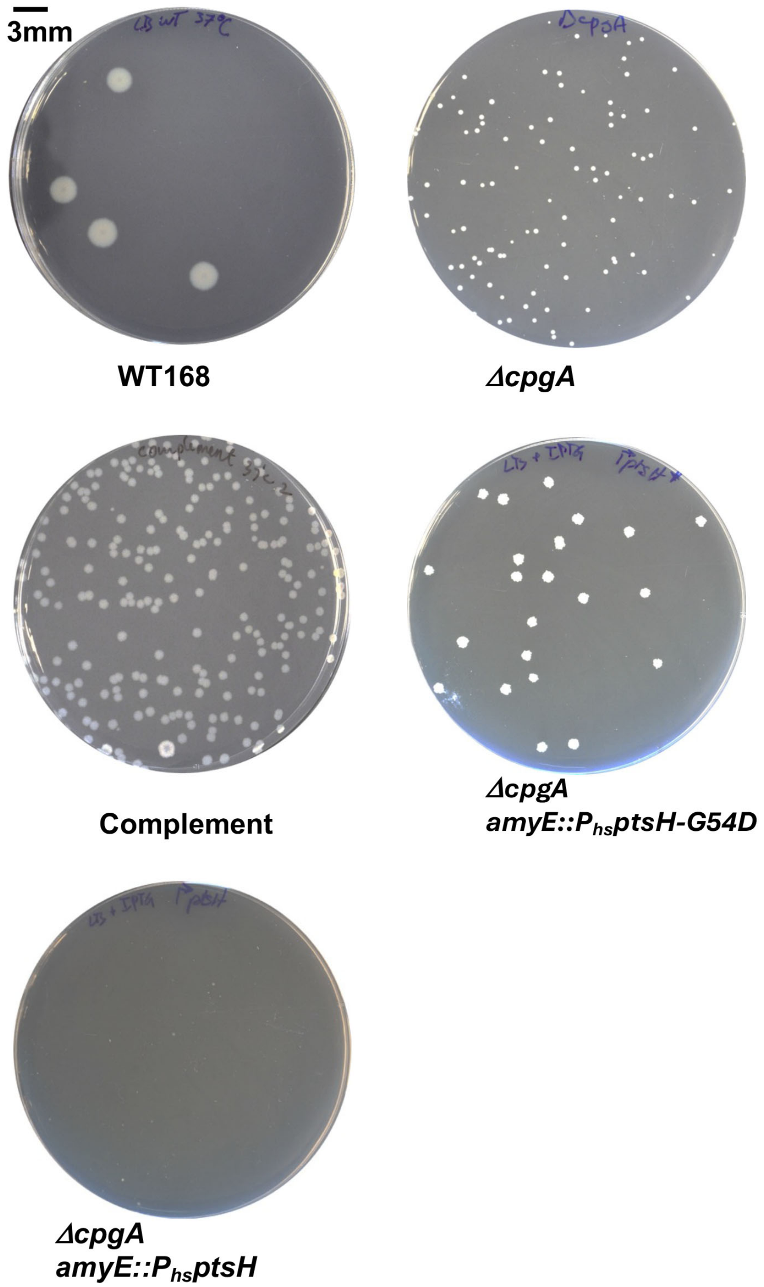

Fig S1: Colony size for various strains. All strains were aerobically grown in LB till mid-log phase and 0.1 mL of culture was spreader on to either LB or LB containing IPTG. All the plates were incubated at 37 °C. Images were taken after incubation. Note: complement strain shows *at-locus cpgA* expression using pMUTIN4 plasmid. Phs-constructs integrated at the *amyE* locus were expressed with IPTG.

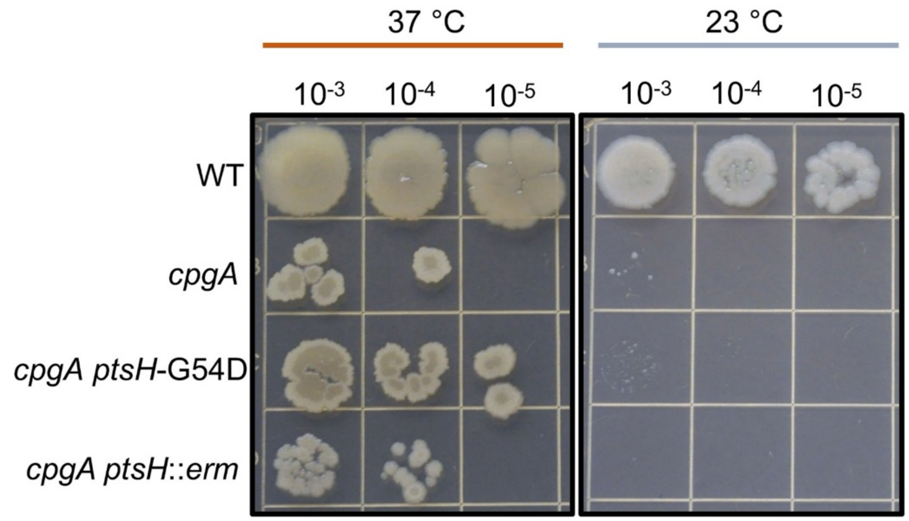

Fig S2: The *ptsH*-G54D mutations is unable to suppress the cold sensitivity of Δ*cpgA*. All cultures were grown aerobically in LB broth at 37 °C to a mid-log phase and were serially diluted and 10 μL of culture was spotted on to LB agar plates and allowed to dry in a laminar hood. These plates were subsequently incubated for colony growth at either 37 °C or at 23 °C. Images were captured after 30 hr.

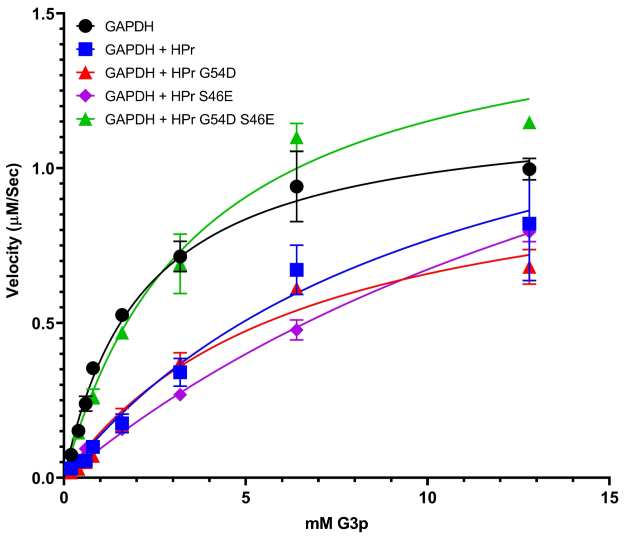

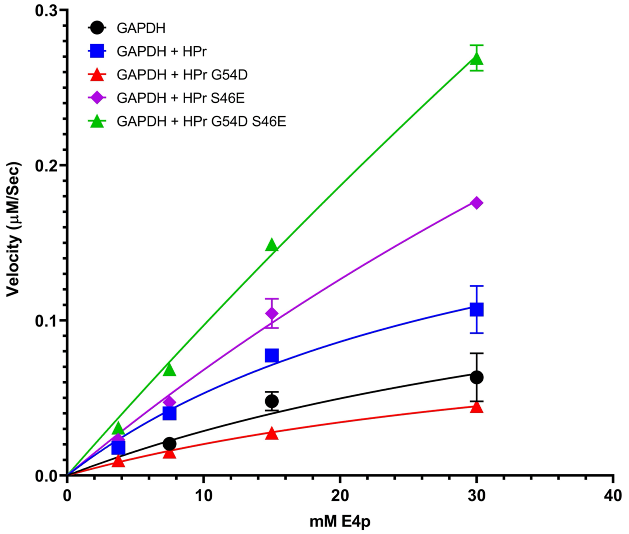

Fig S3: Kinetics of GapA (GAPDH) using G3P and E4P. GAPDH activity was measured using an arsenate-based buffer, NAD^+, and different substrate concentrations. Arsenate is included in place of inorganic phosphate since the spontaneous hydrolysis of arsenoesters makes the forward reaction irreversible. Levels of NADH were monitored spectrophotometrically at 340 nm, and the reaction velocity was calculated using linear regression in GraphPad Prism. Michaelis-Menten enzyme kinetic parameters were calculated using non-linear regression in GraphPad Prism. Values are the mean and standard error of the mean of at least three replicas.

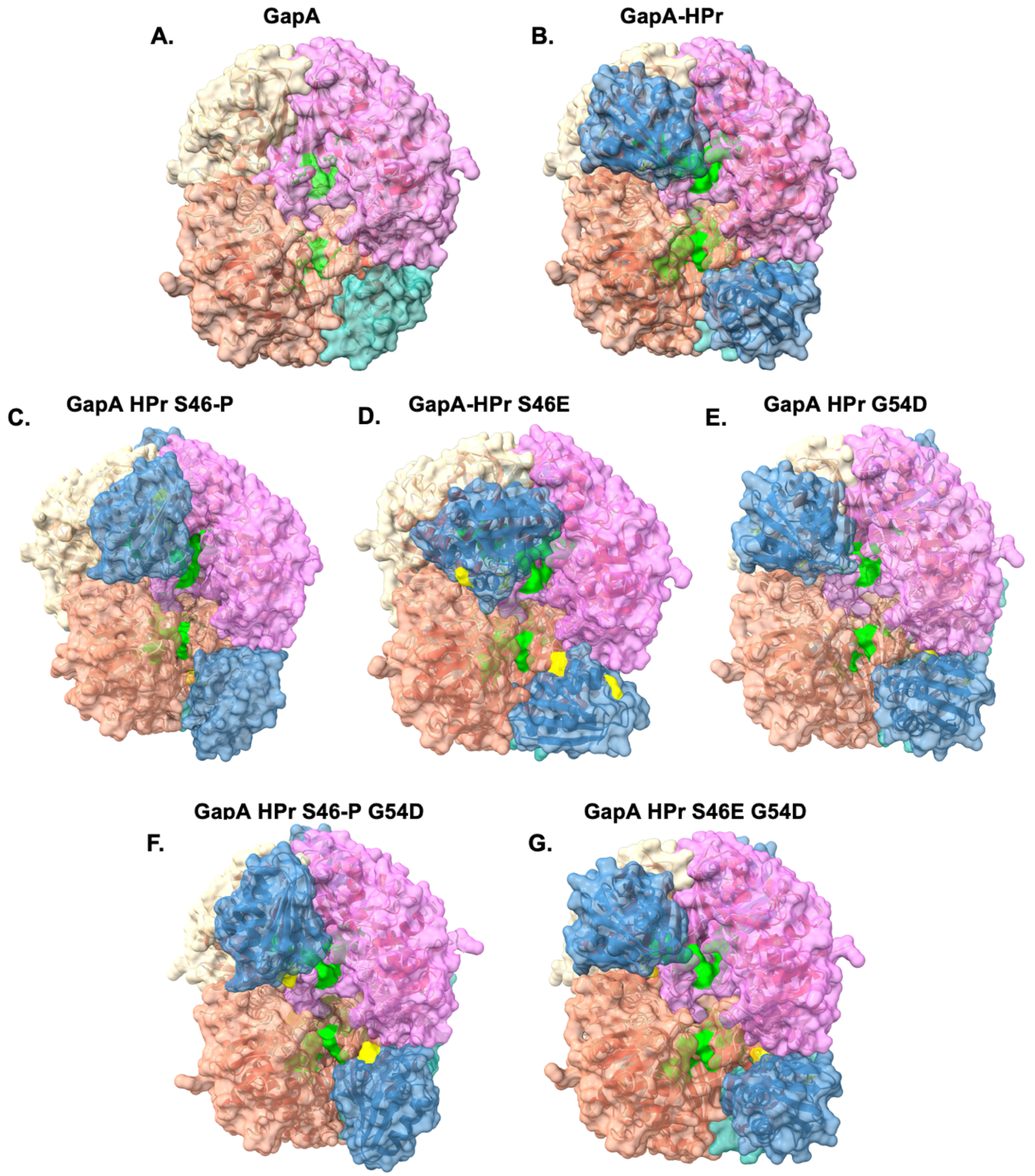

Fig S4: **Phosphorylation of HPr S46 and G54D substitution alter the HPr interaction with GapA**. AlphaFold 3 (4) was used to predict the structure and interaction of GapA tetramer (A) with four HPr monomers (blue) corresponding to: HPr (B), HPr-S46-P (C), HPr-S46E (D), HPr-G54D (E), HPr-S46-P,G54D (F) and HPr-S46E,G54D (G). GapA residues involved in G3P binding are green, and the HPr G54/D54 position is in yellow. GapA monomers are beige, pink, cyan, and dark Salmon. The complexes were categorized as high confidence interactions based on the

predicted template modeling (pTM) and interface template modeling (ipTM) scores (Table S3). Each HPr protein interacted with 3 of the 4 GapA protomers, with a total interaction surface of >800 $Å^2$ (Table S3), corresponding to ~18 to 25% of the HPr surface area, and is greater than the contact area (~700 $Å^2$) of HPr-S46-P with CcpA in a repression complex (6).

The interactions between HPr (B) and HPr-G54-D (E) with the GapA tetramer were very similar in predicted orientation. In contrast, the HPr-S46-P protein had a different predicted orientation that more effectively precludes access to the active site (C). The HPr-S46E phosphomimetic also led to predicted reorientation of the HPr protein (D), but not to the same extent as HPr-S46-P. HPr-S46E,G54D (G) is predicted to revert to a conformation more closely resembling that of the unmodified HPr, whereas HPr-S46-P,G54D (F) is predicted to have intermediate properties. These modeled structures are consistent with the hypothesis that HPr can form a stable complex with the GapA tetramer, this interaction can modulate access to the active site, and modifications at the Ser46 and Gly54 positions may affect the functional consequences of this interaction.

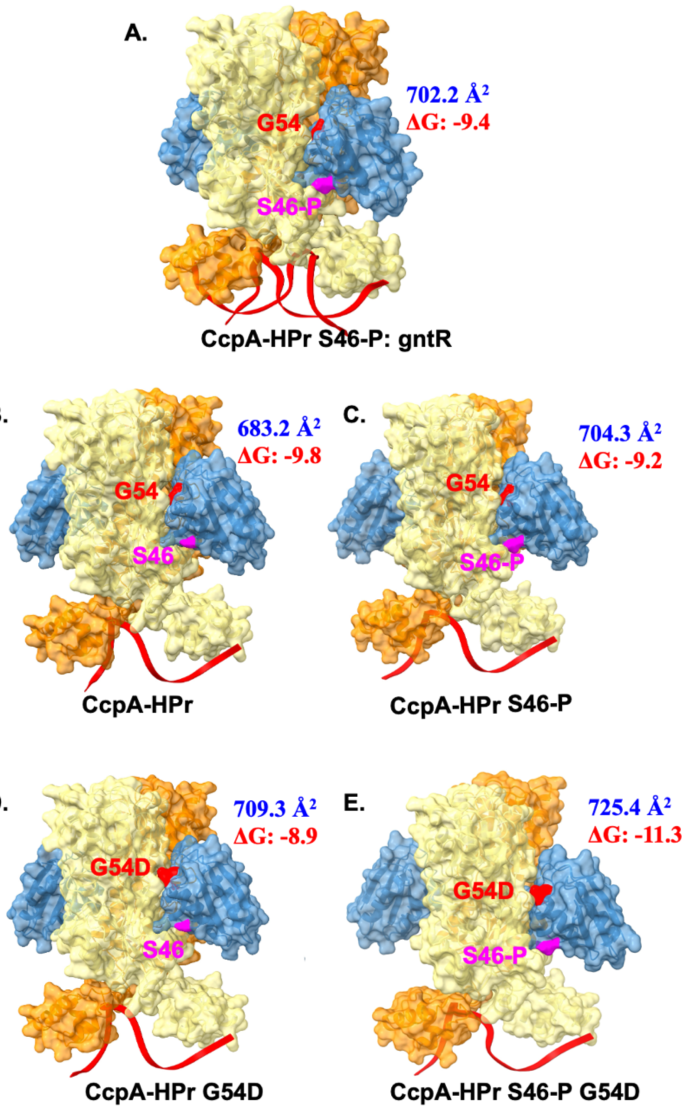

Fig S5: HPr G54D increases the HPr interaction interface with CcpA. (A) Structure of the CcpA-HPr-S46-P complex bound to the *gntR* promoter (Structure accession number 3OQN; (6)), and (B-E) AlphaFold 3 prediction of the structure and interaction of CcpA with HPr (B), HPr-S46-P (C), HPr-G54D (D), HPr-S46-P,G54D (E). The known CcpA-HPr-S46-P structure and the predicted structures were assessed by calculating the interface area and free energies from individual subunit-subunit interactions using the PDBe Protein Interfaces, Surfaces, and Assemblies (PDBe PISA) server (5). The interface area (blue) and free energies (red) represent the average of the total interface interaction between each HPr monomer and the CcpA subunits.

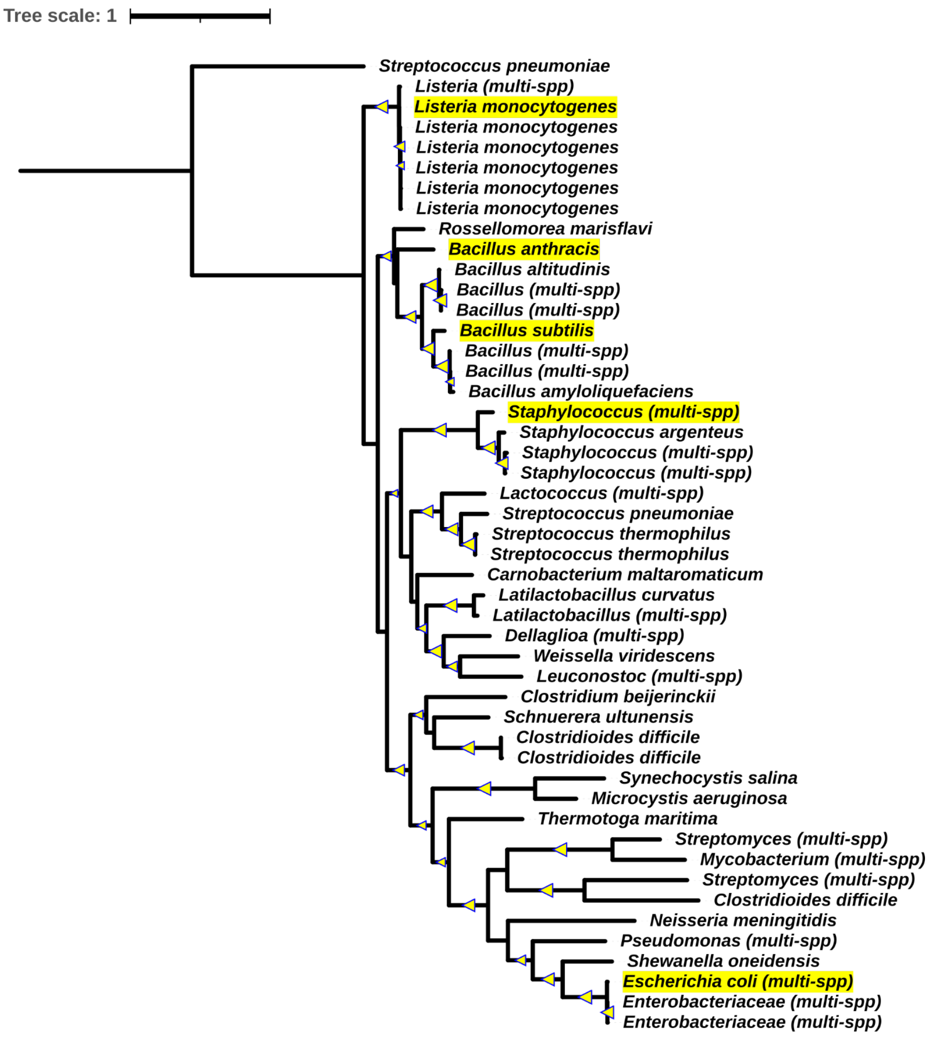

Fig S6: Maximum likelihood (ML) phylogeny inferred from CpgA family protein multiple sequence alignment. Sequences were aligned using MUSCLE (7). The ML phylogeny was constructed with RAxML, using the GAMA substation model and 100 bootstrap replicates. The tree was edited using the iTOL web server (https://itol.embl.de/) and rooted at the midpoint, with branch lengths reported in substitutions per site. Yellow triangle size denotes branches with bootstrap values of 50%-100%. Sequence alignments for the highlighted strains are shown in Fig. S7.

```
Bat             MP--------------------EGKIVKALSGFYYVQHE----EGITQCR
Bsu             MP--------------------EGKIIKALSGFYYVLDESEDSDKVIQCR
Eco             MS--------------------KNKLSKGQQRRVNANHQ----RRLKTSK
Lmo             MILEQYGITSFFKEQKIAATSSYGRVTAVFRDYYRVITEN---EEFLASL
Sau             MK--------------------TGRIVKSISGVYQVDVN----GERFNTK
                *                      .::         .  :

Bat             G-----------------------------------RGVFRKNK--ITPL
Bsu             G-----------------------------------RGIFRKNK--ITPL
Eco             EKPDYDDNLFGEPDEGIVISRFGMHADVESADGDVERCNIRRTI--RSLV
Lmo             K-----------------------------------RGNFYELSSTSLPT
Sau             P-----------------------------------RGLFRKKK--FSPV
                                                    *  : .

Bat             VGDQVVFQADNP------SEGYVLEVFDRKNELVRPP--------IANVD
Bsu             VGDYVVYQAEND------KEGYLMEIKERTNELIRPP--------ICNVD
Eco             TGDRVVWRPGKPAAEGVNVKGIVEAVHERTSVLTRPDFYDGVKPIAANID
Lmo             VGDFVEISGD----------LQILSVLERKTVFSRMNKDSAEQLIAANFD
Sau             VGDIVEFDVQNI------NEGYIHQVYERKNELKRPP--------VSNID
                .** *                 :  : :*.. : *           .*.*

Bat             QAILVFSAVEPDFNPGLLDRFLVLIEYHNIKPIICISKMDLVDEK-MRET
Bsu             QAVLVFSAVQPSFSTALLDRFLVLVEANDIQPIICITKMDLIEDQDTEDT
Eco             QIVIV-SAILPELSLNIIDRYLVACETLQIEPIIVLNKIDLLDDE-GMAF
Lmo             YALIVM-SLNHDFNLNRLERYLTVAWDSGATPIIILTKADLVEDL-SFY-
Sau             TLVIVMSAVEPNFSTQLLDRFLVIAHSYQLNARILVTKKDKTPIE-KQFE
                  ::*  ::  .:.   ::*:*.        . * :.* *

Bat             VEAYANDYREMGYD--VLFTSINTSESIDILKPFLE-GCVSVVAGQSGVG
Bsu             IQAYAEDYRNIGYD--VYLTSSKDQDSLADIIPHFQ-DKTTVFAGQSGVG
Eco             VNEQMDIYRNIGYR--VLMVSSHTQDGLKPLEEALT-GRISIFAGQSGVG
Lmo             AQ----QLEAVAYGVPAYYVDNLSHHGFEALESDLKPNSTLILLGSSGVG
Sau             INELLKIYENIGYE--TEFIGND--DDRKKIVEAWS-AGLIVLSGQSGVG
                 :      . :.*   .   .    ..   :          :. *.****

Bat             KSSMLNVLRP-ELELKTNDISSHLGRGKHTTRHVELIAIG-SG-LVADTP
Bsu             KSSLLNAISP-ELGLRTNEISEHLGRGKHTTRHVELIHTS-GG-LVADTP
Eco             KSSLLNALLGLQKEILTNDISDNSGLGQHTTTAARLYHFPHGG-DVIDSP
Lmo             KSSFINSLAG-TNLMKTAGIREDDSKGKHTTTHREMHLLS-NGWIVIDTP
Sau             KSTFLNHYRP-ELNLETNDISKSLNRGKHTTRHVELFERQ-NG-YIADTP
                **:::*        :  * *  .  . *:***   .:      .*  :  *:*

Bat             GFSSLDFIDIEVEDLTYCFPELKEASQYCKFRGCTHLSEPKCAVKAAVEE
Bsu             GFSSLEFTDIEEEELGYTFPDIREKSSSCKFRGCLHLKEPKCAVKQAVED
Eco             GVREFGLWHLEPEQITQGFVEFHDYLGLCKYRDCKHDTDPGCAIREAVEE
Lmo             GMREFGV-GFNQAGLETTFSDVEELAEGCRFHDCSHTQEPNCAVQAALED
Sau             GFSALDFDHIDKDEIKDYFLELNRYGETCKFRNCNHIKEPNCNVKHQLEI
                *.  : .  ::   :   * :..     *:::.* *  :* * ::  :*

Bat             GKITEYRYKNYKQFVEEIRE------------RKPRY-------
Bsu             GELKQYRYDHYVEFMTEIKD------------RKPRY-------
Eco             GKIAETRFENYHRILESMAQ------------VKTRKNFSDTDD
Lmo             GTLTMQHYENWLKLQREMAYHARKNSPALARQERDRWKVI-QKS
Sau             GNIAQFRYDHYLQLFNEISN------------RKVRY-------
                * :   ::.:: .:  .:               : *
```

Fig. S7: Multiple sequence alignment of CpgA orthologs. Selected CpgA homologous proteins from Gram-positive bacteria were aligned with *E. coli* RsgA using the MUSCLE alignment software (7). *Bacillus* GTPase active site and a critical residue (K177 at left end of line 6) are highlighted in green. Residues in *E. coli* RsgA that do not align with CpgA and were deleted in the truncated version (Eco(trunc)) used for heterologous expression in *B. subtilis* are highlighted in pink. Bsu: *B. subtilis* sp 168, Eco: *E. coli*, Sau: *S. aureus*, Lmo: *L. monocytogenes*, and Bat: *B. anthracis* is used to indicate orthologs.

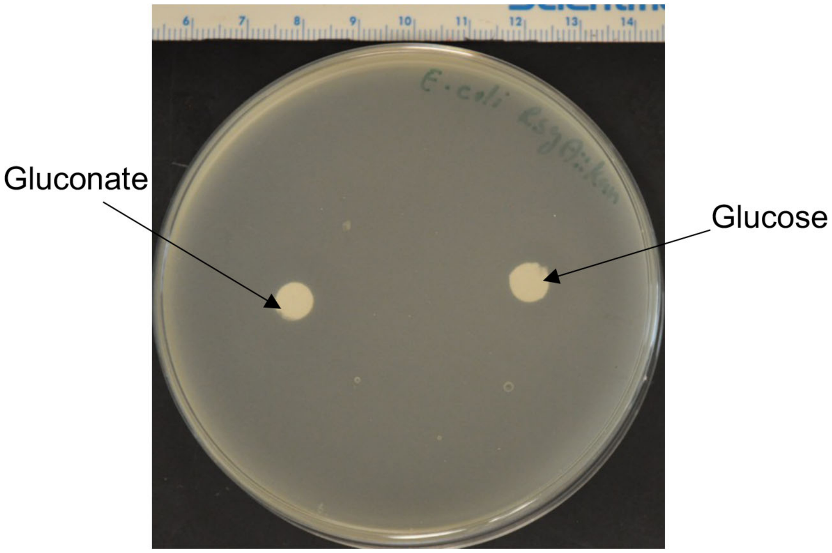

Fig S8: *E coli rsgA*::*kan* is not sensitive to glucose or gluconate.
E coli rsgA gene knockout (*rsgA*::*kan*) cells were grown aerobically to a mid-log phase and then 0.1 mL of culture was added to a 4 mL of soft MH agar (0.75 % agar) and was mixed and overlayed onto hard MH agar (1.5 % agar). Gluconate disk (16 μL of 25 % gluconate) and glucose disk (10 μL of 50 % glucose) were added and plates were incubated overnight at 37 °C. Images were captured after 18 hr.

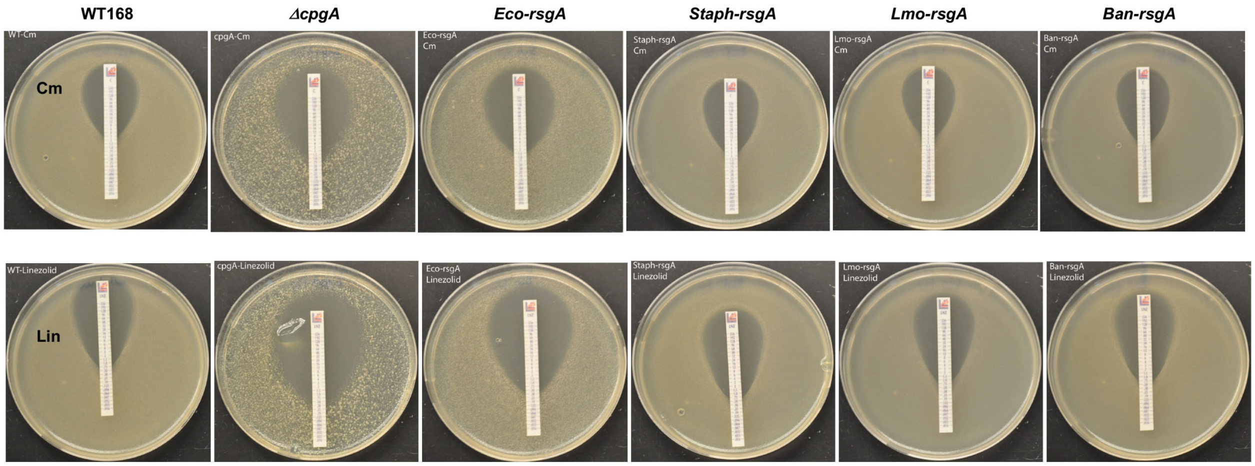

Fig S9: Chloramphenicol and linezolid MIC determination by antibiotic strips.
All cultures were grown aerobically to a mid-log phase and then 0.1 mL of culture was added to a 4 mL of soft LB agar (0.75 % agar) and was mixed and overlayed onto hard

LB agar (1.5 % agar). Antibiotic eStrips were aseptically added and plates were incubated overnight at 30 °C. Zone of clearance as well as zone of less density was noted around disk (n=3). Images were captured after 18 hr.

SI References

1. Sachla AJ, Helmann JD. 2019. A bacterial checkpoint protein for ribosome assembly moonlights as an essential metabolite-proofreading enzyme. Nat Commun 10:1526.
2. Meinken C, Blencke HM, Ludwig H, Stülke J. 2003. Expression of the glycolytic gapA operon in *Bacillus subtilis*: differential syntheses of proteins encoded by the operon. Microbiology (Reading) 149:751-761.
3. Eschenfeldt WH, Lucy S, Millard CS, Joachimiak A, Mark ID. 2009. A family of LIC vectors for high-throughput cloning and purification of proteins. Methods Mol Biol 498:105-15.
4. Abramson J, Adler J, Dunger J, Evans R, Green T, Pritzel A, Ronneberger O, Willmore L, Ballard AJ, Bambrick J, Bodenstein SW, Evans DA, Hung CC, O'Neill M, Reiman D, Tunyasuvunakool K, Wu Z, Žemgulytė A, Arvaniti E, Beattie C, Bertolli O, Bridgland A, Cherepanov A, Congreve M, Cowen-Rivers AI, Cowie A, Figurnov M, Fuchs FB, Gladman H, Jain R, Khan YA, Low CMR, Perlin K, Potapenko A, Savy P, Singh S, Stecula A, Thillaisundaram A, Tong C, Yakneen S, Zhong ED, Zielinski M, Žídek A, Bapst V, Kohli P, Jaderberg M, Hassabis D, Jumper JM. 2024. Accurate structure prediction of biomolecular interactions with AlphaFold 3. Nature 630:493-500.
5. Krissinel E, Henrick K. 2007. Inference of macromolecular assemblies from crystalline state. J Mol Biol 372:774-97.
6. Schumacher MA, Sprehe M, Bartholomae M, Hillen W, Brennan RG. 2011. Structures of carbon catabolite protein A-(HPr-Ser46-P) bound to diverse catabolite response element sites reveal the basis for high-affinity binding to degenerate DNA operators. Nucleic Acids Res 39:2931-42.
7. Edgar RC. 2004. MUSCLE: multiple sequence alignment with high accuracy and high throughput. Nucleic Acids Res 32:1792-7.
